# Constructing a metadata knowledge graph as an atlas for demystifying AI pipeline optimization

**DOI:** 10.3389/fdata.2024.1476506

**Published:** 2025-01-07

**Authors:** Revathy Venkataramanan, Aalap Tripathy, Tarun Kumar, Sergey Serebryakov, Annmary Justine, Arpit Shah, Suparna Bhattacharya, Martin Foltin, Paolo Faraboschi, Kaushik Roy, Amit Sheth

**Affiliations:** ^1^AI Institute, University of South Carolina, Columbia, SC, United States; ^2^Hewlett Packard Enterprise Labs, Houston, TX, United States

**Keywords:** AI pipeline metadata, graph learning, graph recommendation, AIMKG, metadata knowledge graphs, AI pipeline optimization

## Abstract

The emergence of advanced artificial intelligence (AI) models has driven the development of frameworks and approaches that focus on automating model training and hyperparameter tuning of end-to-end AI pipelines. However, other crucial stages of these pipelines such as dataset selection, feature engineering, and model optimization for deployment have received less attention. Improving efficiency of end-to-end AI pipelines requires metadata of past executions of AI pipelines and all their stages. Regenerating metadata history by re-executing existing AI pipelines is computationally challenging and impractical. To address this issue, we propose to source AI pipeline metadata from open-source platforms such as Papers-with-Code, OpenML, and Hugging Face. However, integrating and unifying the varying terminologies and data formats from these diverse sources is a challenge. In this study, we present a solution by introducing Common Metadata Ontology (CMO) which is used to construct an extensive AI Pipeline Metadata Knowledge Graph (AIMKG) consisting of 1.6 million pipelines. Through semantic enhancements, the pipeline metadata in AIMKG is also enriched for downstream tasks such as search and recommendation of AI pipelines. We perform quantitative and qualitative evaluations on AIMKG to search and recommend relevant pipelines to user query. For quantitative evaluation, we propose a custom aggregation model that outperforms other baselines by achieving a retrieval accuracy (R@1) of 76.3%. Our qualitative analysis shows that AIMKG-based recommender retrieved relevant pipelines in 78% of test cases compared to the state-of-the-art MLSchema-based recommender which retrieved relevant responses in 51% of the cases. AIMKG serves as an atlas for navigating the evolving AI landscape, providing practitioners with a comprehensive factsheet for their applications. It guides AI pipeline optimization, offers insights and recommendations for improving AI pipelines, and serves as a foundation for data mining and analysis of evolving AI workflows.

## 1 Introduction

The rapid evolution of artificial intelligence (AI) has led to significant advancements in techniques, necessitating continuous knowledge updates. The submission rates at conferences such as NeurIPS, which constitutes several thousand papers, demonstrate the rapid growth and competitiveness in AI research. Furthermore, there are several versions of generative models being released for various tasks which are difficult to keep track of. The rate of invention of new AI methods also introduces a challenge of suitable model selection for a given task and dataset. The success of AI methods has also led to the development of larger and more complex models to tackle various challenges, due to which training of AI models has become increasing challenging with its increasing complexity. To improve training efficiency, AutoML methods (He et al., 2021; Karmaker et al., 2021) have been introduced for optimizing models and hyperparameter tuning. MLFlow (Zaharia et al., 2018) and MLSchema (Publio et al., 2018) use a model-centric approach for metadata logging, requiring separate instances for each trained model for a given pipeline, say, entity extraction from health records. Openlineage (Hariharan et al., 2024) tracks data lineage through its lifecycle. However, AI pipeline development also includes stages such as dataset selection, preprocessing, feature engineering, and deployment. For reproducibility, metadata logging must encompass all stages, not just model selection and tuning. A comprehensive framework is needed to track all stages, executions, models, and datasets to solve a given AI task.

The Common Metadata Framework (CMF; Koomthanam et al., 2024) addresses this by serving as a pipeline-centric metadata logging system that captures metadata for all stages, executions, models, datasets, and metrics in an integrated manner, enabling the search for the optimal execution path. While CMF provides a holistic approach to metadata logging, a robust framework is required to facilitate AI pipeline optimization. Such optimization can be achieved by leveraging logged metadata to recommend past successful pipeline executions as a seed, reducing the overall experimentation runs. This recommendation requires detailed metadata of numerous pipelines executed, capturing the interactions and dependencies (e.g., input/output datasets, parameters, and configurations) of each stage. Generating such metadata by executing pipelines is not feasible as it demands time and computational resources. On the other hand, open-source platforms such as Papers-with-code (2018), OpenML (Vanschoren et al., 2014), Hugging Face (2016), and Kaggle (2010) expose metadata of already executed pipelines which can be leveraged. To enable metadata interoperability from diverse sources, they must be integrated. However, it poses challenges such as differing nomenclature, data structure variations, and lack of component semantics to understand context and perform reasoning on the entities.

To address these challenges, we introduce the Common Metadata Ontology (CMO), built on the foundations of CMF's pipeline-centric approach. CMO is an unifying schema to integrate metadata from these diverse sources to construct AI pipeline Metadata Knowledge Graph (AIMKG) that enables search and recommendation of relevant AI pipelines for optimization. Knowledge graphs (KG) provide a deeper understanding of relationships and enable context-aware recommendations by capturing both explicit and implicit connections. CMO supports such semantic and multimodal properties which are computed while constructing AIMKG. For example, AIMKG can identify the shared semantics between pipelines for *object detection* and *3d instance segmentation*, recognizing them as vision-based tasks even without explicit naming and facilitate reasoning. As a downstream application, we develop a search and recommender system that demonstrates the potential of AIMKG to recommend relevant pipelines for optimization. The recommender system provides explainable recommendations and ensures reproducibility by providing source information of AI pipelines. The specific contributions of the study are as follows:

1. Proposing Common Metadata Ontology with a pipeline-centric view to integrate and aggregate the metadata mined from diverse sources. CMO supports semantic properties and multimodal properties such as text and embedding vectors.
2. Construction of the first of its kind AIMKG using CMO that serves as an atlas to navigate the ever-growing AI field.
3. Enriching the AIMKG with additional knowledge and by computing semantic properties.
4. Introducing custom heuristic ranking function to recommend relevant pipelines using task, dataset, or model.
5. Introducing a custom aggregation model to generate graph embeddings that enable AI pipeline recommendation for natural language queries.

Conventional knowledge graphs capture relationships among concepts or entities and their semantic properties. For example, Linked Open Data Cloud (Musto et al., 2016) and DSKG (Färber and Lamprecht, 2021) capture semantic relationships among datasets. In contrast, AIMKG consists of process graphs that capture procedural interactions of entities in the context of training and execution, such as how datasets and models combine to produce performance metrics (e.g., dataset + model metrics, model weights). AIMKG follows the traditional semantics of entities and extends it further to process graphs. This procedural representation is a notable contribution not typically found in traditional knowledge graphs.

## 2 Related work

With the growth of AI models (Menghani, 2023; Mathew et al., 2021; Shrestha and Mahmood, 2019; Mohammadi et al., 2024; Berahmand et al., 2024), several frameworks have been proposed to enable the search and discoverability of these models and architectures. DeepSciKG (Kannan et al., 2020) project proposes a mechanism to create and query knowledge graphs to represent multimodal information from AI publication metadata, i.e., code, pseudocode, tables, images, and diagrams in addition to text/equations in publications. STM-KG (Brack et al., 2021) proceeds along similar lines to demonstrate how science, technology, and medicine papers can be automatically mined to automatically populate a scientific concepts knowledge graph and drive a “citation recommender.” ML Schema (Publio et al., 2018) proposed a model-centric ontology to formalize only OpenML data. Humm and Zender (2021) developed an ontology to represent ML metadata to organize and store limited number of tasks (15 as compared to 5 k in our study). AI-KG (Dessì et al., 2020) generated AI knowledge graph from published papers consisting of 330k research publications with 14 M triples that describes five types of entities (tasks, methods, metrics, materials, and others). AIMKG consists of combination of published papers (1 million) and also user-recorded metadata from OpenML and HuggingFace.

The extraction of knowledge from publicly available resources remains an active and dynamic area of research. Notably, the GraphGen4Code (Abdelaziz et al., 2021) approach has emerged as a comprehensive toolkit for constructing knowledge graphs from program code, effectively facilitating subsequent endeavors to address the creation of AutoML pipelines utilizing such knowledge graphs (Helali et al., 2022). These endeavors complement our own study, which leverages existing public repositories and published research to infer relations between AI pipeline entities. By constructing a knowledge graph, our approach aims to solve downstream tasks within the field.

In literature, there has been a consistent effort to recommend datasets for scientific problems, e.g., DataHunter (Färber and Leisinger, 2021) and DataFinder (Viswanathan et al., 2023). Croissant is a high-level format for machine learning datasets that combines metadata, resource file descriptions, data structure, and default ML semantics into a single file for downstream tasks (Akhtar et al., 2024). SIGMOD (Kumar et al., 2023) recommends datasets, models, processing steps etc. along with pipeline lineage. Similarly, other studies such as Müllner et al. (2022) use the history of AI pipelines to recommend datasets and models to solve new tasks allowing sharing of these artifacts among multiple pipelines. In our study, we integrate and aggregate multiple data sources instead of focusing on a particular data source. Specifically, our study focuses on pipeline optimization, knowledge discovery, search, and recommendation through mining metadata from diverse open sources. HuggingGPT (Shen et al., 2023) is a collaborative system that consists of an LLM as the controller and numerous expert models as collaborative executors from HuggingFace. It uses LLM-based chat interface to recommend models for tasks from different domains. We plan to incorporate an LLM interface similar to HuggingGPT in the future while focusing recommending pipeline that includes task, dataset, dataset preprocessing steps, model, metrics, and hyperparameters.

Several techniques such as CASH (Thornton et al., 2013; Guo et al., 2019) and NAS (Elsken et al., 2019) have been proposed for model optimization and hyperparameter tuning. However, our study distinguishes itself by extending beyond the confines of solely addressing problem-dataset or model-hyperparameter relationships. Instead, it delves into capturing intricate associations among models, datasets, and tasks, encompassing their hierarchical connections.

## 3 AI pipeline Metadata Knowledge Graph construction

### 3.1 Data sources

In this study, we collect AI pipeline metadata from Papers-with-Code, OpenML, and HuggingFace to construct AIMKG. The data availability of each source can be found in Table 1. In the future, we also plan to incorporate the metadata from Kaggle.

**Table 1:** Availability of pipeline metadata from open-source platforms.

**Entities**	**PWC**	**OpenML**	**HF**	**Kaggle**
# Pipelines	1 Million +	10 million +	267,000	160 k
# Tasks	4 k	1.6 K	41	200+
# Datasets	12 k	3.4 k	56,000	173 K
# Models	2 k	16 k	267,000	NA

#### 3.1.1 Papers-with-Code

Papers-with-Code provides extensive metadata for research papers and associated code repositories, encompassing over 1 million entries at the time of this paper submission. The metadata covers various components and stages of AI pipelines described in the papers. Through their API, Papers-with-Code offers metadata including PDF URLs, GitHub repository links, task details, dataset information, methods employed, and evaluation metrics and results. While not all stages of metadata are available for every paper through the API, the information can still be obtained by referring to the research papers and their code repositories.

#### 3.1.2 OpenML

OpenML provides metadata on ML pipelines logged by users, offering detailed information on tasks, datasets, flows, runs with parameter settings, and evaluations. OpenML encompasses eight major task types executed on various datasets, resulting in 1,600 unique tasks. For each task, the most recent 500 runs have been collected which amounts to a total of 330,000 runs.

#### 3.1.3 HuggingFace

Huggingface is a model hub that offers users access to numerous pretrained models. It covers a wide range of tasks, including domains such as computer vision, natural language processing, tabular data, reinforcement learning, and multimodal learning. Huggingface provides model-centric information, along with datasets and evaluations, enabling the construction of complete pipelines. At the time of paper submission, ~270,000+ pipelines have been collected from HuggingFace.

### 3.2 Common metadata ontology

**Figure 1:**
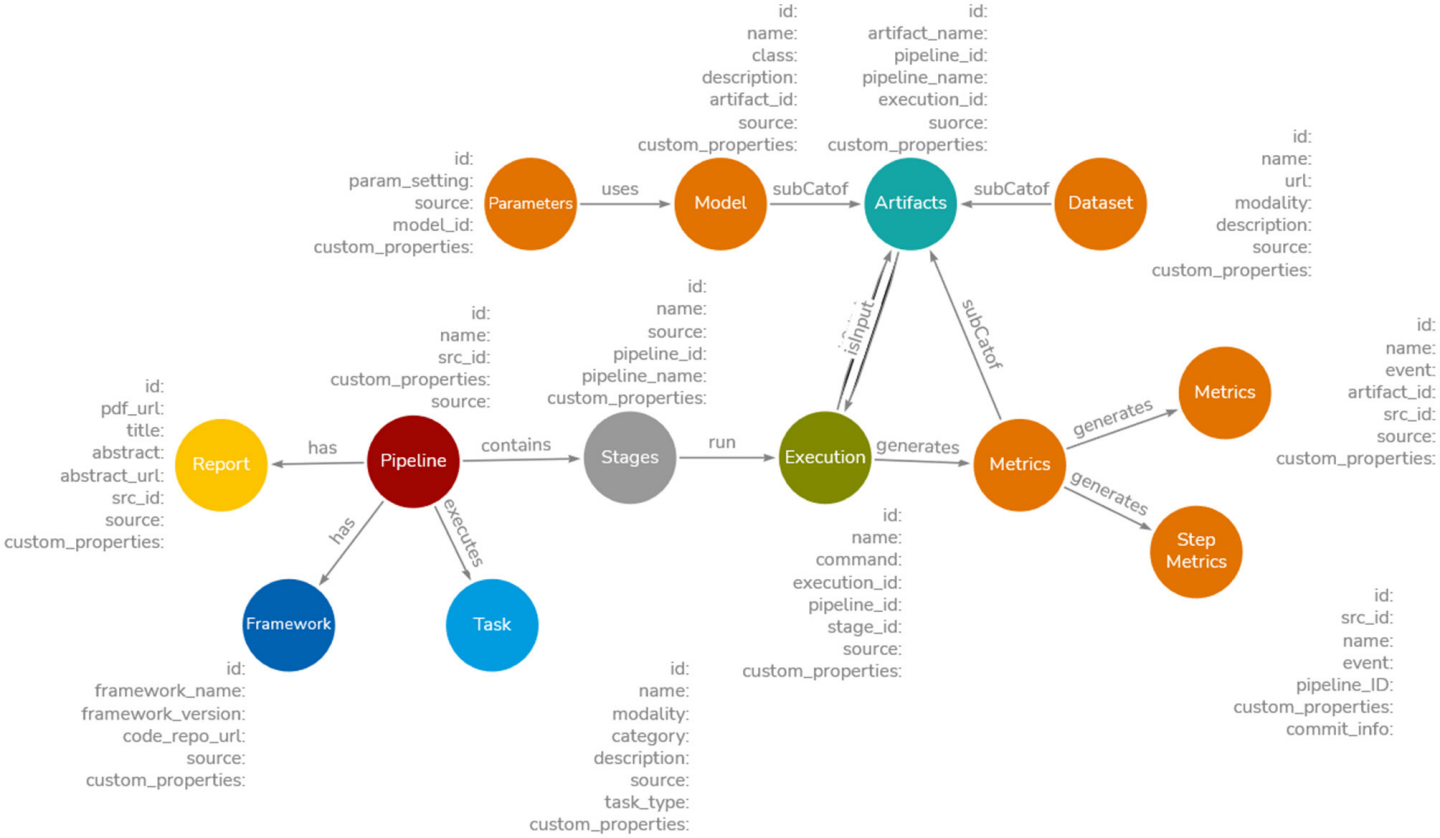
Overview of proposed Common Metadata Ontology. The detailed version of CMO with properties can be found at Venkataramanan (2024). The ontology consists of a pipeline node executing several stages such as data preprocessing, train, test, and validation. Each stage can have several Executions. Each Execution can use a Model and Dataset to produce Metrics and trained model weights. The Task captures a broader view of pipelines such as anomaly detection or demand forecasting. The code base is captured by Framework node and any published report or papers are present in Report node.

The metadata from these sources follows different data structures and nomenclatures. For example, the concept “Model” is referred to as “Methods” in Papers-with-Code, “Flow” in OpenML, and “Model” in Hugging Face. We propose the Common Metadata Ontology (CMO), a unifying schema to integrate diverse data structures from Papers-with-Code, OpenML, and Hugging Face. Built on the Common Metadata Framework (CMF; Koomthanam et al., 2024), CMO ensures interoperability of metadata, enabling knowledge discovery, search, and reasoning capabilities. The overview of CMO is shown in Figure 1. The novel features of CMO are as follows: (i) following a pipeline-centric approach, similar to CMF, to capture multiple experimentation runs for each stage (train, test, validation) with parameter settings, facilitating the identification of the best execution path; (ii) modularity that allows distributed experiments and parallel logging of pipeline metadata, enabling seamless metadata capture across different teams and machines; (iii) support for additional semantic and statistical properties that can be extracted, computed, or generated from entity names (e.g., identifying tasks as image-based, text-based, or audio-based); and (iv) support for multimodal properties, including text and vector embeddings of entity names, to enable keyword and approximate search. A detailed overview of CMO and its properties can be found in Venkataramanan (2024). MLSchema (Publio et al., 2018) and MLFlow (Zaharia et al., 2018) adopt a model-centric approach. When building a pipeline, say, entity extraction from semi-structured electronic health records and testing it with multiple models, MLSchema and MLFlow require creating several instances “one for each model” to record metadata. In contrast, CMO allows all models, variations, hyperparameters, metrics, and datasets to be documented as a single instance, facilitating a scaleable and flexible metadata recording process by taking a holistic view of the entire pipeline. Hence, CMO builds upon the principles of CMF.

### 3.3 Problem statement

The goal is to design a correspondence mapping function *F* that maps the Entity-Relationship-Attributes present in the relational database of three data sources to the Nodes-Relationships-Properties of CMO. Each data source *D* = {*E, R, A*} where *E* = {*e*_1_, *e*_2_, …, *e*_*i*_} represent the set of entities, *R* = {*r*_1_, *r*_2_, …, *r*_*j*_} represent the relationship between *e*_*i*_ and *e*_*j*_, *A* = {*a*_1_, *a*_2_, …, *a*_*n*_} represents the set of attributes for any entity *e*_*i*_. First, each data source *D* is mapped to a graph *G* using a mapping function *f* : *D* → *G*.*G* = {*V, M, K*} where *V* = {*v*_1_, *v*_2_, …, *v*_*i*_} represents the set of vertices, *M* = {*m*_1_, *m*_2_, …, *m*_*j*_} represents the set of edges between vertices, and *K* = {*k*_1_, *k*_2_, …, *k*_*n*_} represents the set of properties of the vertices. The graph *G* of each data source consists of inherent entities, their associations, and properties present in relational database. Then, we compute a correspondence function *F* : *G* → *KG* that maps the elements from graph *G* by computing, extracting, or generating necessary information. *KG* = {*N, R, P*} where *N* = {*n*_1_, *n*_2_, …, *n*_*i*_} are the set of nodes, *R* = {*r*_1_, *r*_2_, …, *r*_*j*_} are the set of relationships between nodes, and *P* = {*p*_1_, *p*_2_, …, *p*_*n*_} are the set of properties of the nodes. For each *G*, *F* : *V* → *N, F* : *M* → *R* and *F* : *K* → *P*. Finally, the AIMKG is constructed as (*KG*1 ∪ *KG*2 ∪ *KG*3).

### 3.4 AIMKG construction and enrichment

#### 3.4.1 Construction

**Figure 2:**
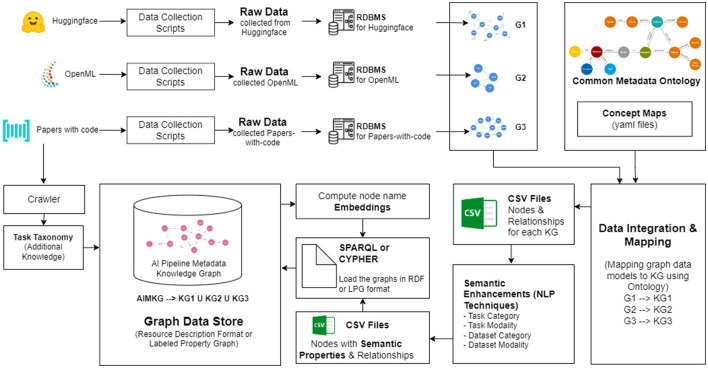
Overview of AIMKG construction. The data collected from Papers-with-code, OpenML, and Huggingface are translated into their relational database formats and then to their graph data models G1, G2, and G3. Then, they are mapped onto CMO. The pipeline metadata undergoes semantic enhancements before loaded as AIMKG.

The algorithm for construction of AIMKG is described in Algorithm 1, and the system architecture is shown in Figure 2. We collect metadata from Papers-with-Code, OpenML, and HuggingFace and represent the data using relational database. The metadata, in the relational database format *D*, is then converted into graph data models *G* through a mapping function *f* : *D* → *G* to analyze the inherent graph structure of each data source. To align the concepts of graph *G* to the concepts in the CMO, we implement a correspondence mapping function *F* : *G* → *KG*. The mapping function *F* consists of a predefined set of mappings of concepts presented in Supplementary Table 1. While specific nodes, relationships, and properties in *G* directly correspond to CMO, additional elements are computed, extracted, or generated by analyzing indirect associations among the entities in each data source. For example, while mapping OpenML data to CMO, the concept node Hyperparameters and Metrics needs to be computed from attributes of Runs given by OpenML. Since these are computed nodes, the relationships need to be computed by studying the associations between tables in relational database provided by OpenML. The mapping of entities from the data sources to CMO can be found in Supplementary material. Currently, AIMKG exists as both Resource Description Format (RDF) and Labeled Property Graph (LPG), and the results are presented in Section 6.

**Algorithm 1:**
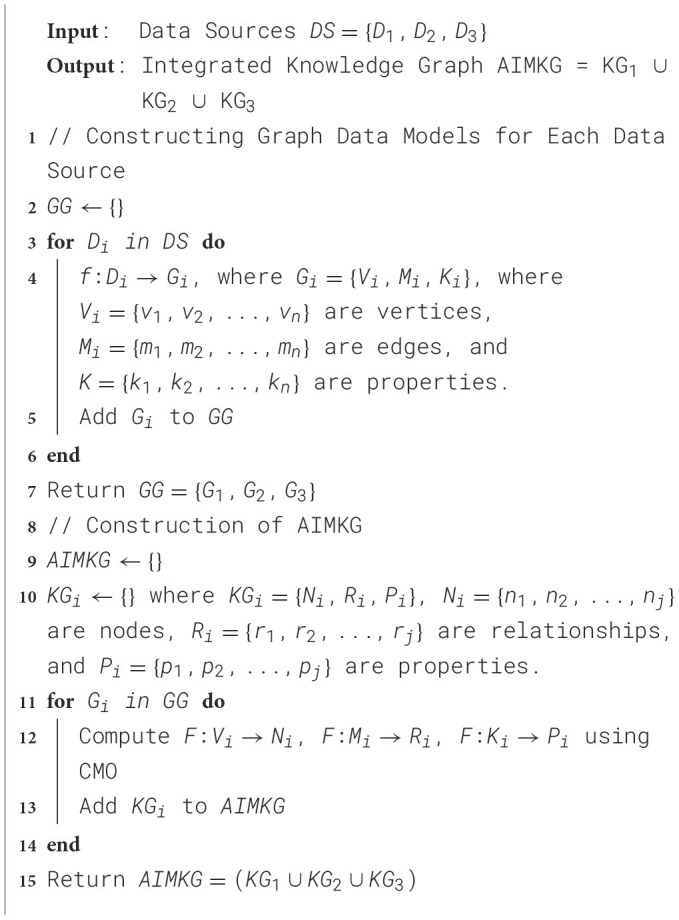
Construction of AIMKG.

#### 3.4.2 AIMKG enrichment

To enable advanced search and recommendation capabilities, we compute semantic properties for pipeline entities, specifically tasks, and datasets. We aim to identify required semantic properties for other nodes in the future work. These semantic properties capture implicit knowledge about entities, providing valuable insights. For instance, the semantic property *modality* identifies the visual nature of tasks such as *object detection* and *video instance segmentation*, even if not explicitly stated. Similarly, capturing task *categories* such as segmentation, classification, or regression clarifies the nature of tasks and aids in organizing and categorizing pipelines by problem type.

##### 3.4.2.1 Rule-based entity extraction

To identify the modality and category of tasks, we utilize a rule-based entity extraction approach. An extensive vocabulary is curated that includes synonyms for each modality and category, such as dialogue, translation, and text for the text modality, and terms such as classification and summarization for categories. Task names and descriptions are analyzed to assign modality and category. The main modalities we consider are Image, Text, Audio, Video, and Multimodal. While this information is available in Papers-with-Code and Huggingface, OpenML presents a challenge as task names are generated by combining task type and dataset name, which obscures modality. To address this, we analyze dataset entities in OpenML, marked as nominal or numeric, to infer task modality. Although the method is straightforward and reliable, using a manually curated vocabulary may introduce biases and limit scalability. Future research will focus on developing more scalable, automated approaches that mitigate biases and enhance robustness.

##### 3.4.2.2 Graph-based labeling of dataset modalities

For the datasets, we determine only *modality* because *category* varies for every pipeline as per the task. For example, MS-COCO dataset can be used for detection, segmentation, or localization. The dataset from all these data sources does not contain sufficient information such as description to identify the modality. Therefore, we study the association between the task and dataset nodes to label *modality* for each dataset. The calculation of dataset modality is as follows:


(1) $$\begin{array}{l} Mod\left(D_{1}\right)=Mod\left(T_{1}\right)\cup Mod\left(T_{2}\right)\cup ...\cup Mod\left(T_{n}\right) \end{array}$$

Where *D*_1_ is the dataset, *T*_1_ to *T*_*n*_ are the associated tasks, and *Mod*(*T*_1_) represents the *modality* computed for a given task.

##### 3.4.2.3 External knowledge

AIMKG also incorporates additional knowledge from various sources to enhance its semantic properties. We crawl task hierarchy information from Papers-with-Code, comprising of three levels: The first level represents task areas such as computer vision, natural language processing, or speech. The second level groups tasks into categories such as segmentation, classification, or detection. Finally, at the leaf nodes, we find the specific tasks provided by Papers-with-Code through their API. This hierarchical structure adds valuable knowledge to AIMKG, enabling a more comprehensive understanding of different task domains.

#### 3.4.3 Node embeddings

To facilitate recommendations or approximate searches, we also compute and store embeddings for the names of tasks, datasets, models, and pipelines. A sentence transformer, *all-mpnet-base-v2* (SBERT Documentation, 2023), with default embedding size 768 was used to create embeddings. The computation of embeddings can be extended to other components of the pipeline as needed. These embeddings, along with the semantic properties, are used in similarity metric calculation to rank relevant recommendation described in the following section. The embeddings are computed and added after standing up AIMKG, allowing flexibility with different models.

## 4 AI pipeline search and recommendation

In certain cases, the exact pipeline the user is searching for can be found in AIMKG. However, it is not always the case. We propose two different recommender systems to search and recommend relevant pipelines to user input query that can seed the experimentation.

### 4.1 Relevant pipeline recommendation using custom heuristics

In this section, we propose a recommender system that enables user to query a relevant pipeline based on its entities such as tasks, datasets, models, or combinations of them. Currently, we develop a custom heuristic ranking metric for tasks, datasets, and models to identify similar pipeline as these three entities are most indicative of a pipeline.

#### 4.1.1 Problem formulation

For a given task *t*_*i*_, dataset *d*_*i*_, or model *m*_*i*_, rank the tasks *T* = {*t*_1_, *t*_2_, ...*t*_*n*_}, datasets *D* = {*d*_1_, *d*_2_, ...*d*_*n*_}, or models *M* = {*m*_1_, *m*_2_, ...*m*_*n*_} present in AIMKG, respectively, using custom heuristics defined below. Once the most similar entities are identified, identify the pipelines associated with top-ranked items by traversing through the graph. Presently, the pipelines recommended consist of coarse-level entities such as tasks, datasets, models, metrics, frameworks, reports, and code repositories. These custom heuristic functions can be used alone or in combination as required. These recommendations act as a seed, reducing the search space for ML practitioners and minimizing the number of experiments needed to achieve optimal solutions.

#### 4.1.2 Heuristic functions

**Task similarity:** Using the *modality* and *category* properties, we compute similarity for the task nodes in AIMKG as follows:


(2) $$\begin{array}{l} task\text{\_}sim\left(t_{i},t_{j}\right)\\ =\frac{cos\left(e_{i},e_{j}\right)+J\left(name_{i},name_{j}\right)+J\left(mod_{i},mod_{j}\right)+J\left(cat_{i},cat_{j}\right)}{4} \end{array}$$

where *t*_*i*_, *t*_*j*_ are any two task nodes, *e*_*i*_, *e*_*j*_ are task name embeddings, *name*_*i*_, *name*_*j*_ are the task name tokens, *mod*_*i*_, *mod*_*j*_ are task modalities (image, text, audio, etc.), and *cat*_*i*_, *cat*_*j*_ are task categories (detection, summarization, classification, etc.). *J* is the Jaccard similarity, and *cos* is the cosine similarity of embeddings.

**Dataset similarity:** Dataset consists of *modality* calculated using Equation 1. They do not have *category* as a semantic property as a given dataset might be suitable for two task *categories* such as segmentation and detection. Therefore, dataset similarity is calculated as


(3) $$\begin{array}{l} dataset\text{\_}sim\left(d_{i},d_{j}\right)\\ =\frac{cos\left(e_{i},e_{j}\right)+J\left(name_{i},name_{j}\right)+J\left(mod_{i},mod_{j}\right)+U\left(url_{i},url_{j}\right)}{4} \end{array}$$

where *d*_*i*_, *d*_*i*_ are any two dataset nodes, *e*_*i*_, *e*_*j*_ are dataset name embeddings, *name*_*i*_, *name*_*j*_ are the dataset name tokens, *mod*_*i*_, *mod*_*j*_ are dataset modalities (image, text, audio, etc.) of the dataset names, *url*_*i*_, *url*_*j*_ are dataset URLs, and *U* is token-based URL similarity metric that quantifies the degree of resemblance between two URLs.

**Model similarity:** Model similarity is computed using the given semantic property *class* such as CNN and GPT. The *URL* given by the sources is also used as in some sources it aids in capturing the root of the model origin (Example: HuggingFace)


(4) $$\begin{array}{l} model\text{\_}sim\left(m_{i},m_{j}\right)\\ =\frac{cos\left(e_{i},e_{j}\right)+J\left(name_{i},name_{j}\right)+J\left(class_{i},class_{j}\right)+U\left(url_{i},url_{j}\right)}{4} \end{array}$$

where *m*_*i*_, *m*_*i*_ are any two model nodes, *e*_*i*_, *e*_*j*_ are model name embeddings, *name*_*i*_, *name*_*j*_ are model name tokens, and *class*_*i*_, *class*_*j*_ are model classes (transformers, CNN, GRU, etc.).

**Figure 3:**
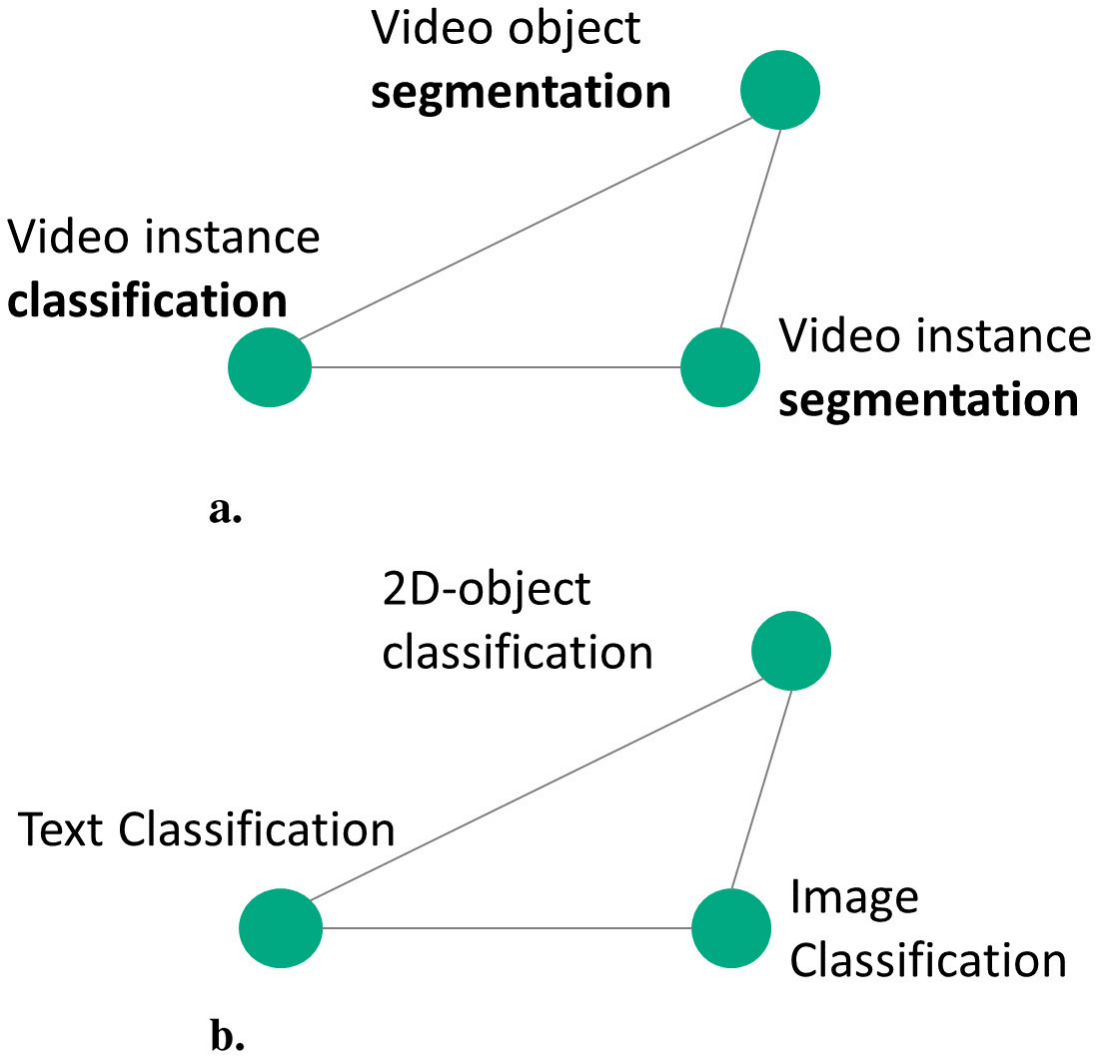
Illustration to show the necessity of embedding and keyword similarity. **(A)** Task category semantics **(B)** Task modality semantics.

We found through empirical experiments that a combination of embedding and keyword similarity offers the best results. For example, embedding similarity captures that “fault” and “anomaly” are synonyms. Simultaneously, in Figure 3A, segmentation tasks must be closer than classification tasks. Similarly, in Figure 3B, image-based tasks need to be closer than text-based tasks. These semantics are not captured by the embedding similarity but through keyword-based similarity of semantic properties computed for pipeline components. The ability to design and implement meta-similarity based on sets and the proximity of textual embeddings is a unique differentiator compared to existing methods such as Achille et al. (2019).

### 4.2 Relevant pipeline recommendation using graph learning

In contrast to the recommender discussed in the section above, this section proposes a custom graph embedding learning model that retrieves relevant pipelines to user input queries given in natural language.

#### 4.2.1 Problem formulation

The goal is to learn a common embedding space for the natural language query and its corresponding pipeline graph to retrieve relevant pipelines. AIMKG graph consists of several pipeline graphs {*P*_1_, *P*_2_, *P*_3_, …*P*_*n*_}. Each *P*_*i*_ = {*N, E*}, where *N* is the set of nodes and *E* is the set of edges. The nodes *N* = {*p, s, e, a, d, m, met, f, r, t*} represent different elements: *p* is pipeline, *s* is stages, *e* is executions, *a* is artifacts, *d* is datasets, *m* is model, *met* is metrics, *f* is framework, *r* is reports, and *t* is tasks. For each pipeline, we have a set of queries *Q*_*i*_ = {*q*_1_, *q*_2_, …, *q*_*n*_}. The goal is to learn a common embedding space for graph embedding *ge*_*i*_ that takes *P*_*i*_ = {*N, E*} as input and query embedding *qe*_*i*_ that takes in one-sentence query *q*_*i*_ as input.

#### 4.2.2 Query generation

Since there is no ground truth information, ChatGPT was used to generate a one-sentence query that describes the pipeline, which can simulate a user query to search for a pipeline. Similar to studies that involve the Retrieval Augmented Generation (RAG) approach (Jadon and Kumar, 2023; Guo and Chen, 2024), we utilized ChatGPT API to generate queries for a given pipeline based on the name and description of node entities such as pipeline, model, task, dataset, and metrics. These generated queries are different from the title of the paper from Papers-with-Code or title of the report or model cards from HuggingFace. The detailed analysis on queries generated can be found in Supplementary material. The following prompt was used to generate one-sentence description for each pipeline:


      PROMPT:
      Generate a vague two-line query summarizing
      the pipeline
      information below, utilizing pipeline
      description, list
      of tasks, list of datasets and list of
      methods. Avoid forming
      the query as a question. Generate these
      queries as if a user
      is searching for a pipeline based on the
      following pipeline
      information. Note, these queries should be
       very different
      from the pipeline name given below.
      Return the query as
      bullets numbered as 1., 2., and 3.
  
      Pipeline Description:
      {data['pipeline_description']}
      Pipeline Name: {data['pipeline_name']}
      List of Tasks:
      {task_string}
  
      List of Datasets:
      {dataset_string}
  
      List of Models:
      {model_string}
  
   


#### 4.2.3 Dataset

For this evaluation, we randomly picked 5,000 pipelines from Papers-with-Code and HuggingFace each, totaling 10,000 pipelines. Only the pipelines with complete information such as model, dataset, task, and metrics were chosen. The pipelines from Papers-with-Code and HuggingFace are more descriptive which is essential for query generation. For example, Papers-with-Code has abstract, dataset description, task description, and so on. Similarly, HuggingFace has model cards, dataset description, and so on. Such descriptive information was not found in OpenML pipelines, and so they are omitted for this evaluation. For each pipeline, on an average of two queries were generated by ChatGPT using the prompt mentioned in Section 4.2.2

#### 4.2.4 Model architecture

**Figure 4:**
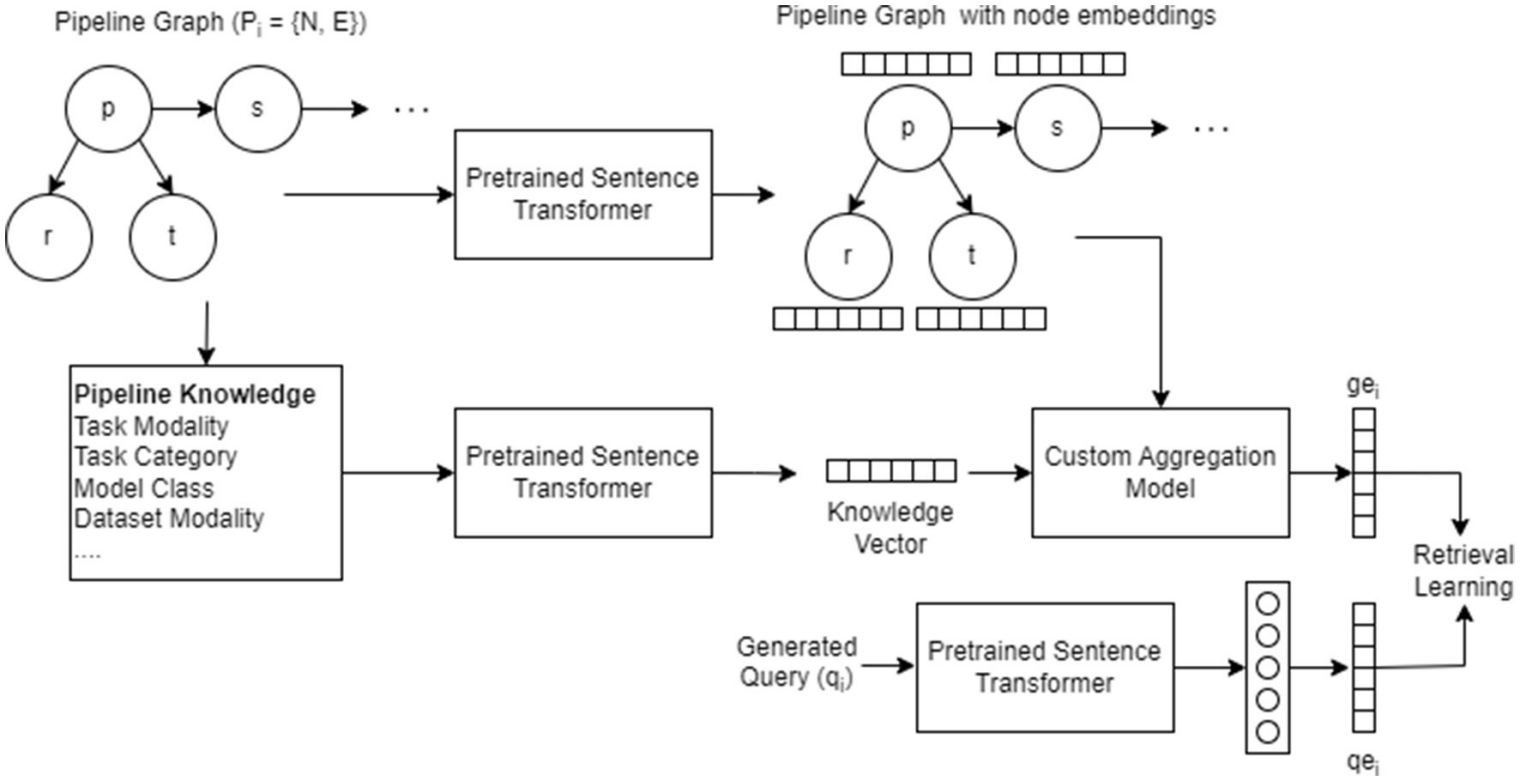
Architecture and workflow of the custom aggregation model utilized to learn the graph embedding.

In this section, we propose a custom model described in Algorithm 2 that utilizes self-attention based aggregation to learn embedding for each pipeline graphs as described in Figure 4. For each node in N, where *N* = {*p, s, e, a, d, m, met, f, r, t*}, the name and description present as text are converted to 768-dimensional embedding using sentence transformer. Using the semantic properties computed for each pipeline graph nodes (Section 3), we create a knowledge string. The knowledge is then passed to a sentence transformer to create embedding for knowledge. Similarly, the generated queries are passed to the sentence transformer to generate respective embeddings. Through empirical analysis, we found that the sentence transformer embeddings perform better compared to a learnable embedding layer with one-hot embeddings.

**Algorithm 2:**
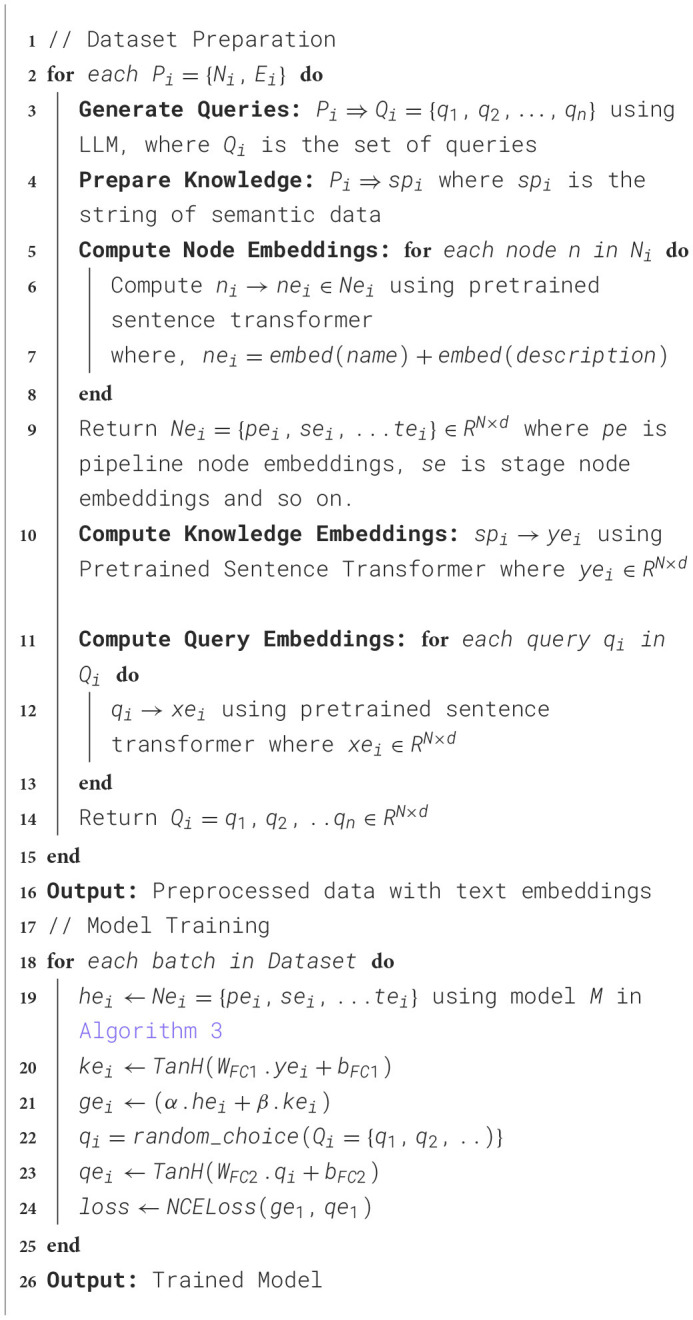
Model training.

Then, these embeddings are given as an input to the self-attention block (Algorithm 3) to generate an intermediate graph embedding of 1024-dimensional vector. Similarly, the embeddings generated for knowledge vector are also transformed into 1024-dimensional vector using a learnable fully connected layer. The learnt embeddings of the nodes and the knowledge vector are combined using a weighted sum to generate final graph embedding *ge*_*i*_. We present the results of the model with and without knowledge embedding in Table 5. The embeddings generated for query vector using sentence transformer embedding are also transformed into 1024-dimensional vector to obtain *qe*_*i*_. The objective function described in Section 4.2.5 trains the model to such that *ge*_*i*_ and *qe*_*i*_ are closer in the embedding space.

**Algorithm 3:**
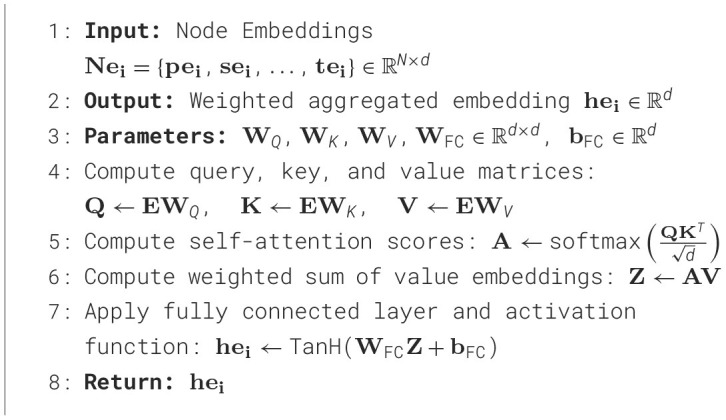
Self-attention based aggregation model (M).

In the case of AIMKG, 1.6 million pipeline graphs follow the graph structure described by CMO (Section 3.2). To add, the textual information present in the nodes holds the most information compared to the graph structure. While the connectivity between models, datasets, tasks, and other nodes of the pipeline is essential to learning an appropriate graph embedding, graph-based models such as graph convolutional neural networks or graph attention neural networks prioritize learning graph topology compared to node features (Section 6.4). For this reason, a custom aggregation model was proposed to learn embedding for each pipeline graph. Table 5 shows the necessity of representing pipelines as a graph.

#### 4.2.5 Objective function

To train the query embedding *qei* and the corresponding graph embedding *gei* to be closer in the embedding space, we use noise contrastive estimation (NCE) loss (Chen et al., 2020). NCE loss has the ability to normalize large probability distributions making it effective for scalable training datasets. The equation for NCE loss is as follows:


$$\begin{array}{c} L_{\text{NCE}}=-\frac{1}{N}\overset{N}{\underset{i=1}{\sum }}(\log\frac{\exp\left(ge_{i}\cdot qe_{i}\right)}{\exp\left(ge_{i}\cdot qe_{i}\right)+\overset{k}{\underset{j=1}{\sum }}\exp\left(ge_{i}\cdot qe_{j}\right)}\\ +\log\frac{\exp\left(ge_{i}\cdot qe_{j}\right)}{\exp\left(ge_{i}\cdot qe_{j}\right)+\overset{k}{\underset{j=1}{\sum }}\exp\left(ge_{j}\cdot qe_{i}\right)}) \end{array}$$

where *N* is the batch size, *ge*_*i*_ and *qe*_*i*_ are the embeddings for the *i*-th instance in the batch, and *k* is the number of negative samples which is *N* − 1 where *i* ≠ *j*.

## 5 Experimentation

In this section, we define evaluation metrics performed to test the robustness of AIMKG and recommendation ability of AIMKG.

### 5.1 AIMKG robustness

To evaluate the reliability of our knowledge graph construction, we employ a statistical technique called bootstrapping (Anirudh and Thiagarajan, 2019). We take a random sample of 75% of the data and utilize it to create a partial knowledge graph using our proposed approach (AIMKG). We repeat this process 10 times to generate 10 distinct knowledge graphs. A robust knowledge graph construction method should show low variance in node properties within partial graphs. We measure variance in node degrees and other distributional properties, comparing these with knowledge graphs built using the MLSchema ontology. The performance improvements are detailed in the result section.

### 5.2 Custom heuristics: qualitative analysis

Through user evaluation, we evaluate the ability of custom heuristic function to rank similar tasks for a given unknown task and return its associated pipelines. Due to the abscence of ground truth, we rely on domain experts to evaluate the relevance of results returned by the recommender. For comparison, a knowledge graph constructed using same data but using state-of-the-art MLSchema ontology is used. Using the custom heuristic function, the tasks in AIMKG and MLSchema-based KG are ranked and associated pipelines are returned to the domain experts for evaluation. The task nodes in AIMKG contain properties such as Name, Modality, Category, and Description. Modality and Category are computed using NLP techniques. In contrast, MLSchema-based task nodes only have properties such as Name, Description, and custom user-reported properties. Therefore, *S*_*mod*_ and *S*_*cat*_ from Equation 2 are always 0 for the recommender that uses MLSchema-based KG whereas *cos*(*e*_*i*_, *e*_*j*_) and *J*(*T*_*i*_, *T*_*j*_) are calculated using the same procedure. We configure the recommender to return the top-k relevant tasks and n pipelines for each task, where k and n are set to 3. We randomly select query tasks from various AI fields from AIMKG (Table 4) and drop the query task node to simulate unknown query task. This ensures the validity of the query task names. Eighteen domain experts aged between 24 and 50 participated in the evaluation study, each assigned 10 query tasks to determine the relevance of the recommendations provided.

### 5.3 Graph embedding learning

#### 5.3.1 Implementation details

We utilize sentence transformer *all-mpnet-base-v2* to generate the text encodings for node features, semantic properties (knowledge), and queries generated by ChatGPT. The default embedding size of 768 was used. Each of these text encodings are transformed into 1024-dimensional vector using a fully connected layer, one for knowledge vector and another for query encodings. We initialize the network with random weights for training. During training, for each pipeline, we randomly sample one query from the available generated queries. The batch size is set to 512. The Adam optimizer (Kingma and Ba, 2014) was used with learning rate 10^-4^ and weight decay set to 1*e*^-5^. We employ early stopping to prevent the model from overfitting and train it for several epochs until it converges.

#### 5.3.2 Evaluation protocols

The custom aggregation model learns a common embedding space to retrieve a process graph given a natural language query. These can be considered two modalities of data, namely, graph and text. Therefore, we evaluate the custom aggregation model described in Section 4.2.4 using retrieval metrics reported by Salvador et al. (2017). For a given query embedding *qe*_*i*_, we retrieve the k closest graph embeddings *ge*_1…*k*_ using cosine similarity and present the results for k = 1, 3, and 5. We perform retrieval evaluation for 1,000 data samples and report results in Table 5. The definition of models reported is as follows:

- **GCN:** A graph convolutional neural network that takes pipeline graph *P*_*i*_ with node encodings *Ne*_*i*_ to generate *ge*_*i*_ obtained using global mean pool of learned node embeddings.
- **GAT:** A graph attention neural network that takes pipeline graph *P*_*i*_ with node encodings *Ne*_*i*_ to generate *ge*_*i*_ obtained using global mean pool of learned node embeddings.
- **Sent_Trans:** Use pretrained sentence transformer to generate *ge*_*i*_ using pipeline name and description. Use query text to generate *qe*_*i*_. Both *ge*_*i*_ and *qe*_*i*_ are 768-dimensional vector as that is the default embedding size for sentence transformers.
- **Sent_Trans_Finetune**: Use *qe*_*i*_ and *ge*_*i*_ from **Sent_Trans** model and transform them into 1024-dimensional vector using a learnable fully connected layer.
- **Custom_Agg:** Model described in Algorithm 3 that takes in node encodings *Ne*_*i*_ for each pipeline graph *P*_*i*_ to learn graph embedding *he*_*i*_ (equivalent of *ge*_*i*_ for this model).
- **Custom_Agg_Knowledge**: Model described in Algorithm 2. It takes the output from **Custom_Agg** *he*_*i*_ and transformed knowledge vector *ke*_*i*_ to learn *ge*_*i*_ = α.*he*_*i*_ + β.*ke*_*i*_ where α and β are learnable weights.

## 6 Result and discussion

### 6.1 AIMKG overview

The statistical overview of AIMKG can be found in Table 2. The AIMKG consisting of knowledge graphs KG1, KG2, and KG3 contains 8 million nodes and 25 million relationships in label property graph (LPG) format. There are ~78 million triples in RDF format which include the vector embeddings computed as properties. There are 11 types of nodes that represent each component of AI pipeline metadata and 13 types of relationships among those entities. Currently, the knowledge graph consists of 1.6 million AI pipelines executed for ~10 k tasks with ~53 k datasets and ~270k models. The knowledge graph is currently growing in size to include more pipelines and additional knowledge. A sample pipeline present in AIMKG is described in Supplementary material. The details of system maintenance and performance are also included in Supplementary material.

**Table 2:** Overall statistics of AIMKG.

**Components**	**Quantity**
# Nodes (LPG)	8 million
# Relationships (LPG)	25 million
# of triples (RDF)	78 million
# Types of nodes	14
# Types of relationships	15
# AI piplines	~1.6 million

### 6.2 AIMKG robustness

The first row in Table 3 shows the results obtained from knowledge graph constructed using MLSchema ontology. It is evident that the variance in node degrees is higher compared to AIMKG. AIMKG demonstrates lower variance, confirming the robustness of the knowledge graph construction scheme. Furthermore, we observe that when using the MLSchema ontology, only 71% of the nodes are part of the largest connected component in the knowledge graph, while the remaining nodes are part of other disconnected components. In contrast, AIKMG includes 93% of the nodes in the largest connected component, indicating a more coherent graph structure for performing downstream tasks.

**Table 3:** Comparative analysis of robustness of AIMKG and MLS-KG.

**KGs**	**Mean variance**	**Median degree**	**Max degree**
MLS-KG	3.465	1	103
AIMKG	2.383	1	389

### 6.3 Relevant pipeline recommendation using custom heuristics

The results of user agreement on the relevance of recommendations provided for query tasks are summarized in Table 4. The goal is to return relevant pipelines for a given query of unknown task. For each areas such as computer vision, natural language processing, audio/speech, and video, 20 query tasks were evaluated. Due to limited number of pipelines in AIMKG, 10 queries were evaluated for multimodal and other areas that includes graphs, reasoning, and game-related learning. In total, 100 queries were evaluated for each recommender. According to domain experts, the recommender utilizing AIMKG achieved relevant results for 78% of the queries, while the MLSchema-based recommender had a lower success rate of 51%. The Cohen's kappa score computed for the subset (25%) of the queries was found to be 0.657, which is considered a substantial agreement between the domain experts on the recommendation relevance. The computed semantic properties utilized by the custom heuristic function (Equation 2) played a significant role in understanding task nature and capturing synonyms. For example, a query for *Dialogue Interpretation* returned *Dialogue Understanding* as a relevant task, showcasing the recommender's ability to recognize synonyms.

**Table 4:** User evaluation study.

**Areas**	**AIMKG**	**MLS-KG**
Computer vision	17/20	8/20
Natural language processing	16/20	10/20
Audio/speech	15/20	11/20
Video	15/20	10/20
Multimodal	6/10	6/10
Other	9/10	6/10
Total	78/100	51/100

Relevance of recommendations produced by AIMKG and MLS-KG recommenders.

In computer vision queries, there was a notable difference in relevance scores due to significant number of challenging queries which did not explicitly mention the word “image.” For example, *3D object detection* and *3D human pose estimation* do not have the word image in it, but they are image-based tasks. Similarly, NLP-based tasks also benefited from semantic enhancements present in AIMKG. Video-based tasks are extensions of computer vision-based tasks that includes temporal factor. Therefore, like computer vision-based queries, a significant amount of video-based queries did not explicitly have the word “video” in it. Some examples include *motion detection* and *human movement detection*. The category other was challenging for both recommenders as the vocabulary curated for these areas is relatively small to identify modalities of these tasks.

When AIMKG is deployed as an open-source platform, it serves as a curated knowledge repository of open-source AI innovations that are searchable, discoverable, and executable. Users can search among 280 k models, 53 k datasets used for 10 k tasks at one place. It is an AI exploration and experimentation platform that hosts, serves, and refreshes state-of-the-art open-source AI innovations. This enables the reproduction of AI pipelines, including data preprocessing, pretraining, fine-tuning, and model deployment, which are impactful across various use cases. The broader practical impacts of AIMKG in fields such as healthcare, finance, and legal for pipeline optimization through relevant pipeline recommendation can be found in Supplementary material.

### 6.4 Relevant pipeline recommendation using graph learning

From Table 5, it can be observed that our **Custom_Agg_Knowledge** model performed the best and **Custom_Agg** performed second best against other baseline approaches. The difference is that the former model utilizes semantic properties computed for various pipeline graph component entities as described in Section 3.4.2. Furthermore, the proposed method demonstrated statistically significant improvements over baseline methods, confirmed by both Friedman's test (*p* < 0.01) and pairwise Wilcoxon signed-rank tests (*p* < 0.01). The results of sensitivity analysis and ablation study can be found in Supplementary material. **GCN** and **GAT** models weigh in more on learning the topological structure of the graphs compared to node features. In AIMKG, all pipeline graphs follow similar graph structure defined by CMO. To add, most information about the pipeline is present as text in the node features. Due to this, the text information gets diluted over graph structure in **GCN** and **GAT** models. As expected, these models have the least retrieval scores. The sentence transformer model was evaluated with and without fine-tuning to test whether pipeline descriptions (abstract, model-card) suffice for relevant retrieval. The fine-tuned model performed better for the HuggingFace and Combined datasets but not for Papers-with-Code, likely due to its detailed description on pipelines already present in abstract. Fine-tuning may have caused embedding instability for Papers-with-Code, while it improved accuracy for HuggingFace and the Combined dataset.

**Table 5:** Retrieval results of models for 1,000 datapoints, reported in percentage.

**Models**	**Papers-with-Code**			**Huggingface**			**Combined**		
	**R1**	**R3**	**R5**	**R1**	**R3**	**R5**	**R1**	**R3**	**R5**
GAT	47.3	65.8	72.2	33	51.8	60.5	44.3	60	66.2
GCN	52.3	66.1	72.5	39	56.9	64.7	48.4	62.8	69
Sent_Trans	82.6	89.3	91.4	25.8	38.5	44.8	57.6	66.8	70.7
Sent_Trans_Finetune	65.4	80	83.2	47.8	65.6	73.2	69.0	79.6	83.6
Custom_Agg	85.9	90.8	92.2	55.9	69.2	73.9	74.8	82.7	85.8
Custom_Agg_Knowledge	**87.1**	**91.5**	**94.1**	**58**	**71.1**	**75.7**	**76.3**	**85.4**	**87.7**

The bold indicates the best performing model.

In summary, the **Sent_Trans** and **Sent_Trans_Finetune** results show that pipeline graphs are essential for effective retrieval, capturing relationships between datasets, models, tasks, and entities. Descriptions from Papers-with-Code and HuggingFace are limited. Traditional graph models such as **GCN** and **GAT** underperformed on AIMKG due to their focus on topology over node features. The proposed custom aggregation model, emphasizing node features, outperformed others with added knowledge-boosting results.

### 6.5 Pipeline optimization

In this section, we present the results on utilizing recommendations from AIMKG to seed the AI pipeline experimentations. Existing work Pedretti et al. (2023) demonstrated the use of novel in-memory accelerator engines to speed-up the inference of tree-based machine learning models for heterogeneous (tabular) data, the most widely used type of data across various industries. We employed seven widely used real-world tabular datasets for binary/multi-class classification and regression problems from research papers. In this section, we demonstrate the improvements in executing the hyperparameter optimization AI pipelines for gradient-boosted trees (XGBoost; Chen and Guestrin, 2016) on several binary and multi-class classification problems from that paper. Concretely, we collected results from hyperparameter optimization pipelines for four datasets (Eye Movement, Gas Concentration, Gesture Phase Segmentation, and Rossmann Stores Sales). We then imported the pipeline performance data into the AIMKG using one of the developed parses and asked it to recommend pipeline configurations for new, previously unseen, similar problems—churn modeling, telco customer churn, and forest cover type. We then used these recommendations from AIMKG to warm-up the Bayesian (TPE—tree-structured Parzen Estimators; Bergstra et al., 2011) hyperparameter optimization. We compared results with the reference results where no warm-up initialization was made. Table 6 shows three datasets. For each dataset, we report observed speed-up (wall time) to optimize hyperparameters of respective models to same or lower loss. In addition, we observed that the final loss was lower compared to experiments without warm-up initialization.

**Table 6:** Result of pipeline optimization achieved using AIMKG recommender.

**Churn modeling**		**TelcoCustomerChurn**		**ForectCoverType**	
**SpeedUp**	**LossDiff**	**SpeedUp**	**LossDiff**	**SpeedUp**	**LossDiff**
8.60	–0.11%	11.84	–0.63%	1.47	–0.02%

### 6.6 Additional attributes of AIMKG

#### 6.6.1 Search using semantic enrichment and graph traversal

**Figure 5:**
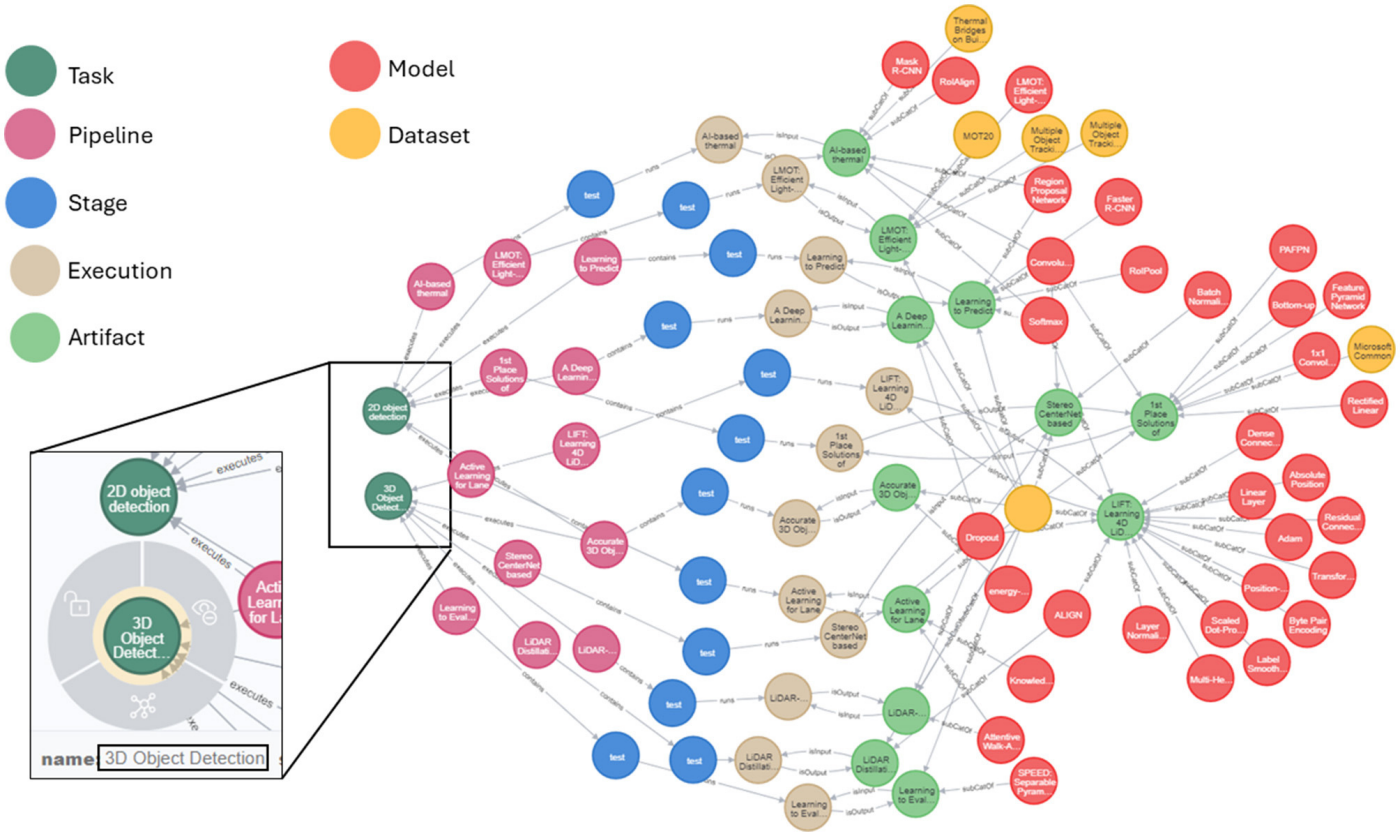
Sample query: list all the image detection pipelines with dataset and evaluations. The tasks “3d object detection” and “2d object detection” are returned by AIMKG even though no explicit mention of “image.” To add, the graph traversed from task to datasets and models to identify models and datasets used for image detection task. More sample queries of pipelines with hyperparameters can be found at Venkataramanan (2023).

We illustrate the potential of AIMKG to perform complex queries that utilizes combination of custom heuristic functions in Section 4.1 and graph traversal to return desired results through Figure 5. We queried recommender to return datasets and models used for *image detection* task. Since this task does not exist in the repository, it identifies *2d-object detection* and *3d object detection* as similar tasks using the heuristic function in Equation 2. Even though the task names did not have explicit mention of the word “image,” they are identified as image-based tasks due to the semantic property *modality*. In addition, the recommender traverses the path from *Task* → *Pipeline* → *Stage* → *Execution* → *Artifact* → *Dataset* and *Task* → *Pipeline* → *Stage* → *Execution* → *Artifact* → *Model* to retrieve models and datasets. More sample queries and their results can be found at Venkataramanan (2023).

#### 6.6.2 Relevant pipeline recommendation using graph traversal

**Figure 6:**
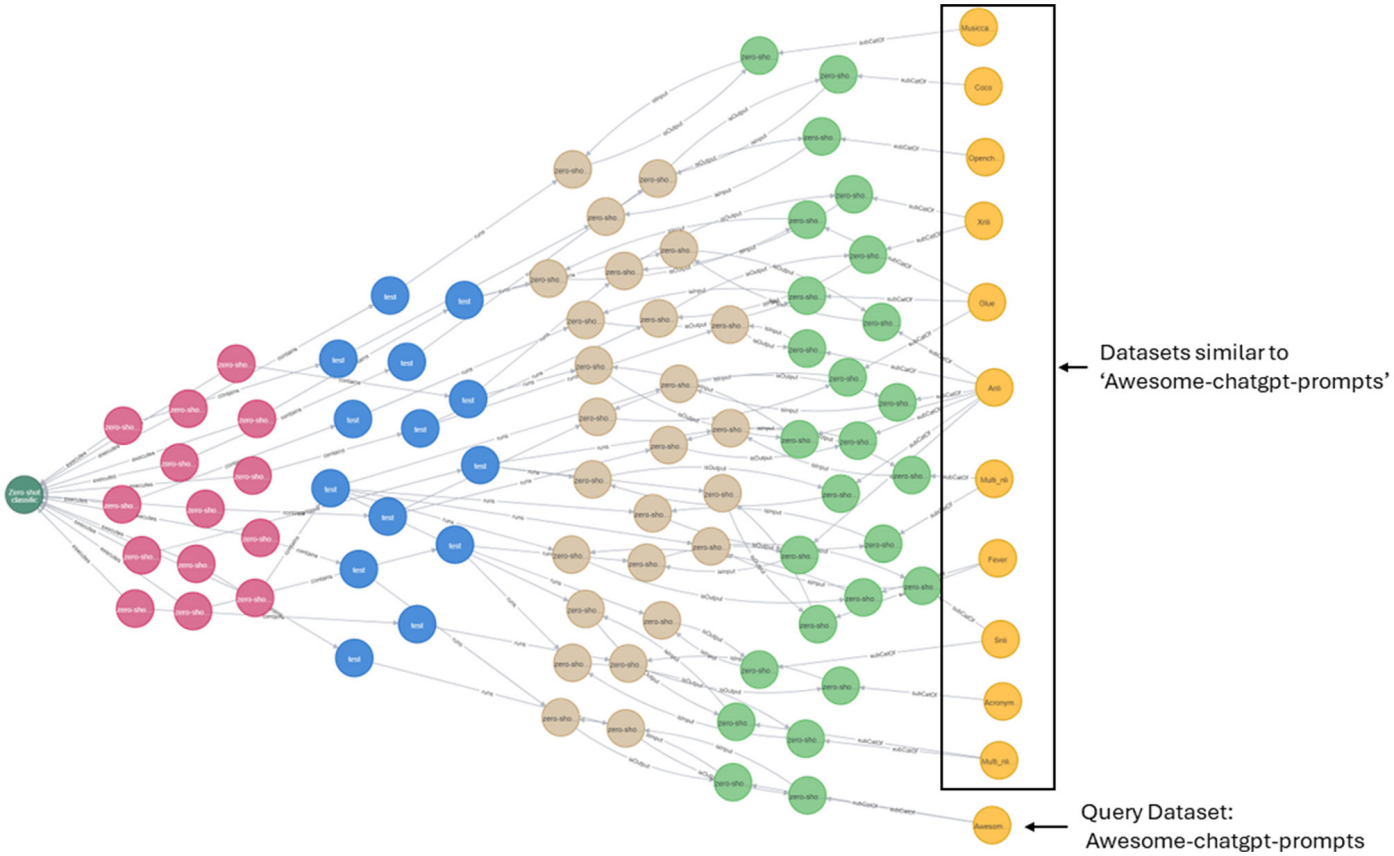
Query: identify datasets similar to *Awesome-chatgpt-prompts*. In this example, similar datasets were identified based on graph traversal. That is, if the datasets are used for the same task, they might be similar in certain aspects.

In addition to Equation 3, similar datasets can also be obtained through graph traversal as shown in Figure 6. The query is to return datasets similar to *Awesome-chatgpt-prompts*. Using the inference that if the datasets are used for the same task, they can be similar in certain aspects, we performed graph traversal query, and the resulting graph is shown in Figure 6. To perform the same query, that is to return similar datasets to a given dataset, other kinds of inferences can be used such as (i) if the datasets are used in the same pipeline, they can be considered; (ii) if the datasets are used in the same pipeline with same model, they can be considered similar and so on.

#### 6.6.3 AIMKG: dynamic AI pipeline knowledge repository

AIMKG is a constantly evolving graph that updates itself periodically by fetching data from Papers-with-Code, OpenML, and HuggingFace. We are also working toward including other metadata sources mentioned in Table 1. This iterative process of periodic updates involves continuous monitoring, ensuring that the graph remains current and reflective of the evolving information landscape. Given that AI domain is ever changing with new models being introduced and manuscripts being published, it is imminent that AIMKG is live and dynamic. We demonstrate the importance of maintaining a live pipeline for AIMKG using the example described in Table 7.

**Table 7:** Comparison of AIMKG at two different timestamps.

**Timestamp 1**
**Input Query**: Models and Pipelines for the task “Question Answering”
**Response:**
Result-1:
Model: (i) BERT, (ii) GELU,
Pipeline: Leveraging Commonsense Knowledge on Classifying False News and Determining Checkworthiness of Claims,
URL: https://arxiv.org/pdf/2108.03731v1.pdf,
Code: none,
Year of publication: 2021
Result-2:
Model: (i) VisualBERT, (ii) Learning Cross-Modality Encoder Representations from Transformers
Pipeline: “A Comparison of Pre-trained Vision-and-Language Models for Multimodal Representation Learning across Medical Images and Reports,”
URL: https://arxiv.org/pdf/2009.01523v1.pdf,
Code: https://github.com/YIKUAN8/Transformers-VQA7,
Year of publication: 2020
...
**Timestamp 2**
**Input Query:** Models and Pipelines for the task “Question Answering”
**Response:**
Result-1:
Model: mulinski/bert-finetuned-squad,
Pipeline: Question Answering using bert-finetuned-squad,
URL: https://huggingface.co/mulinski/bert-finetuned-squad,
Code: https://huggingface.co/mulinski/bert-finetuned-squad/tree/main,
Year of publication: 2023
Result-2:
Model: dantern/xlm-roberta-base-vn-dplat,
Pipeline: Question Answering using dantern/xlm-roberta-base-vn-dplat,
URL: https://huggingface.co/dantern/xlm-roberta-base-vn-dplat,
Code: https://huggingface.co/dantern/xlm-roberta-base-vn-dplat/tree/main,
Year of publication: 2023
. . .

We query AIMKG to return pipelines and models for the task *Question Answering*. Before the integration of most recent models from HuggingFace, AIMKG returned pipelines that were published in 2021 and 2020, respectively. Each of these pipelines used two models in their experimentation. When the same query was ran at a different timestamp, after integrating the most recent models, it returned *bert-finetuned-squad* and *xlm-roberta-base-vn-dplat* as the models used for *Question Answering* along with their pipelines. These models were published in 2023. The result from AIMKG now contains most recent models used for *Question Answering*. This self-updating mechanism not only enhances the graph's comprehensiveness but also ensures that it consistently serves as a reliable and up-to-date resource for users seeking the latest insights and connections within the represented domain. System maintenance and performance details are included in Supplementary material.

#### 6.6.4 Integration of multiple data sources

**Figure 7:**
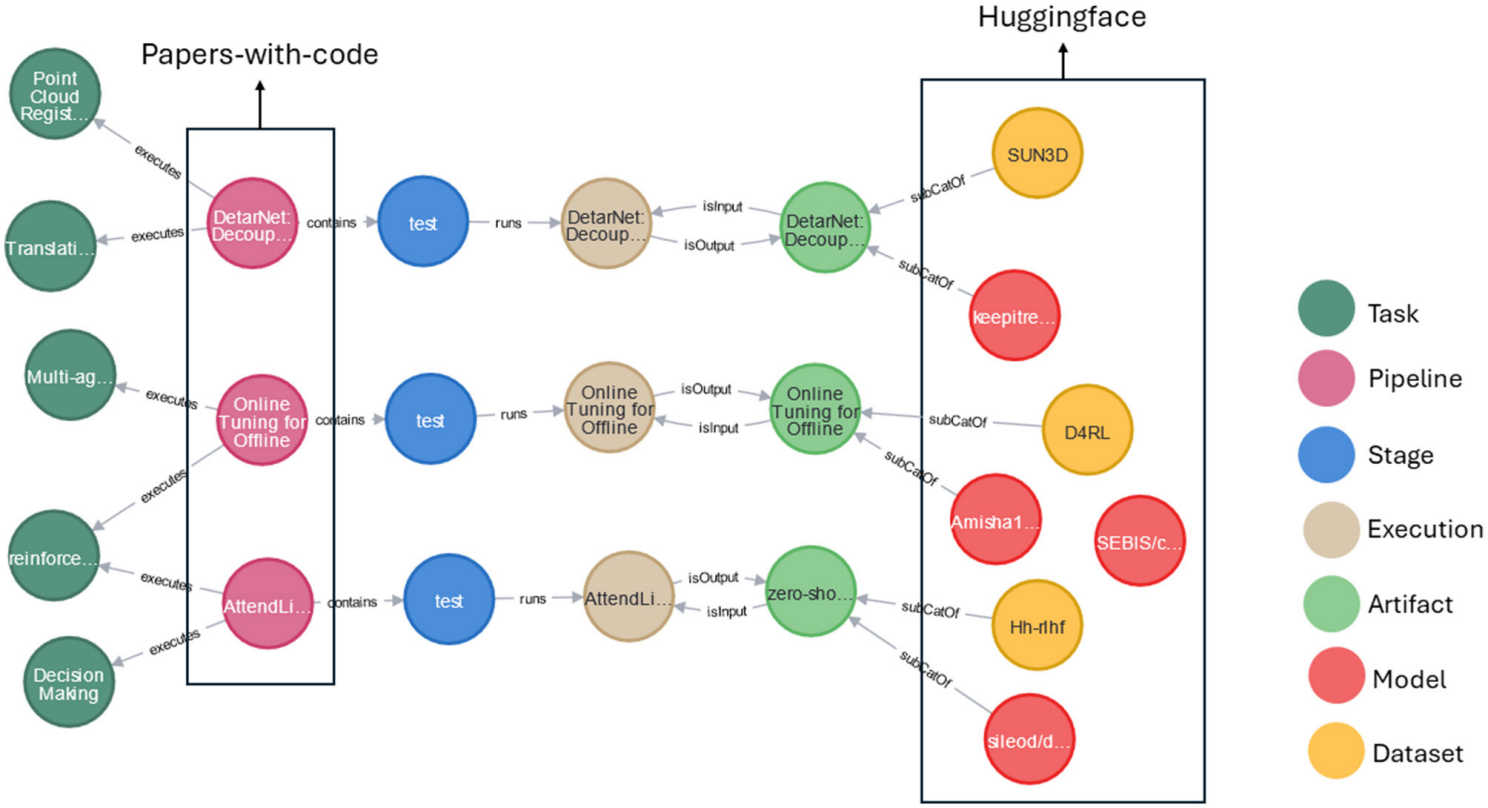
Sample of pipeline completion done by AIMKG by identifying identical concepts from Papers-with-Code and Huggingface.

As mentioned in Section 3, AIMKG consists of pipeline metadata obtained from multiple sources such as Papers-with-Code, OpenML, and HuggingFace. It is worth noting that Papers-with-Code and HuggingFace have information overlap to certain degree. While the overlap has been identified and unified, it has also helped in pipeline completion in certain cases. For example, in Figure 7, AIMKG had pipeline name and report from Papers-with-Code. For these pipelines, the model and dataset information is not available via Papers-with-Code API as they were not recorded by users explicitly. On the other hand, HuggingFace had model and dataset information for these pipelines. By utilizing paper arxiv ID and paper title, our AIMKG construction pipeline identified that these two are from the same pipeline by mapping them to CMO. While there are several such example, a few of them are included in Figure 7 to demonstrate the concept of integration of data sources that can aid in completion of pipeline metadata. Ontologies and knowledge graphs excel at the task of recognizing identical concepts present in various data sources. The ability of ontologies and knowledge graphs to discern shared meanings has enabled AIMKG to identify identical concepts from disparate data contexts.

### 6.7 Comparison with ChatGPT

As Large Language Models (LLMs) have been known to perform several tasks, we compared our AI pipeline recommendation task with ChatGPT-3.5 (OpenAI, 2022). We queried both our AIMKG recommender and ChatGPT to return pipelines executed for the task *Supervised Anomaly Detection* and reported the results in Table 8. Anomaly detection is the task of detecting data instances that significantly deviate from the majority of the data points (Pang et al., 2021). ChatGPT offers an interactive framework for querying, and it returns the models and their papers for common tasks such as 3D-aware image synthesis. The model seems to generate relevant information for familiar tasks from computer vision or natural language processing. However, the model seems to generate non-existent information for less familiar tasks such as anomaly detection or sequence-to-sequence speech generation. ChatGPT was prompted with sample query and sample expected response as mentioned in Table 8. The model generated similar response as given in the prompt. However, the pipelines returned by ChatGPT, in terms of papers, seem to be non-existent in literature. That is, ChatGPT exhibited instances of hallucination, generating information that does not exist in a convincingly realistic manner which is a well-known issue (Zuccon et al., 2023). In the response presented in Table 8, all of GitHub links return 404 when attempted to reach. For all the three recommended papers, the arxiv link is the same and it does not belong to any of the papers recommended. Paper 1 that was recommended with the title “DeepOne-Class: Outlier Detection with Deep Learning” does not exist. This means the model and dataset generated in the response are also in question. For paper 2 and paper 3, the titles are partially correct but papers with exact titles do not exist. Similarly, the datasets mentioned in paper 2 and paper 3 are not the datasets used in the actual paper. It is evident that the recommendations from ChatGPT are not reliable to reproduce or train an AI pipeline. More example responses along with explanations can be found in Supplementary material.

**Table 8:** Comparison of AIMKG recommender with ChatGPT.

**AIMKG results**
**Input query**: Supervised Anomaly Detection
**Response**: Since the exact match is not found, we recommend the following pipelines from relevant task:
**Recommendation 1:**
Similar Task Name: Weakly Supervised Defect Detection
Similarity: 0.4752
Pipline-1: S2D2Net: An Improved Approach For Robust Steel Surface Defects Diagnosis With Small Sample Learning, URL: https://ieeexplore.ieee.org/document/9506405, Git: https://github.com/vikxoxo/S2D2Net
Pipline-2: Mixed supervision for surface-defect detection: from weakly to fully supervised learning, URL: https://arxiv.org/pdf/2104.06064v3.pdf, Git: https://github.com/aws-samples/amazon-sagemaker-edge-defect-detection-computer-vision
...
**Recommendation 2:**
Similar Task Name: Semi-supervised Anomaly Detection
Similarity: 0.4643
Pipline-1: Deep Semi-Supervised Anomaly Detection, URL: https://arxiv.org/pdf/1906.02694v2.pdf, Git: https://github.com/Fraunhofer-AISEC/R2-AD2
Pipline-2: Real-world Anomaly Detection in Surveillance Videos, URL: http://arxiv.org/pdf/1801.04264v3.pdf, Git: https://github.com/ShreyasArthur/AbnormalEventDetection
...
**ChatGPT results**
**Input prompt:** Create a session where you are an AI pipeline recommender. I will request for a pipeline that has been executed in the past. You can use any source you want. For example, when I request to recommend a pipeline for “3d video object tracking”, I expect a response as follows:
Similar Task Name: 3D Object Tracking, Similarity: 0.6083, Piplines: Exploring Point-BEV Fusion for 3D Point Cloud Object Tracking with Transformer, URL: https://arxiv.org/pdf/2208.05216v1.pdf, Model: Transformer, Dataset: KITTI, Github: https://github.com/jasonkks/pttr Piplines: MOTSLAM: MOT-assisted monocular dynamic SLAM using single-view depth estimation, URL: https://arxiv.org/pdf/2210.02038v1.pdf, Model: SLAM, Dataset: KITTI, Github: none
Now give me the pipelines executed for supervised anomaly detection.
**Response:** Similar Task Name: Supervised Anomaly Detection, Similarity: 0.7254
1) Paper Title: “DeepOne-Class: Outlier Detection with Deep Learning,” URL: https://arxiv.org/abs/1802.06360, Model: Deep Neural Networks, Dataset: Credit Card Fraud, NAB, Github: https://github.com/hpzhao/Anomaly-Detection-DeepOneClass
2) Paper Title: “Autoencoder-Based Anomaly Detection,” URL: https://arxiv.org/abs/1802.06360, Model: Autoencoder, Dataset: MNIST, CIFAR-10, Github: https://github.com/abnormal-1/anomaly-detection-resources
3) Paper Title: “LSTM-based Encoder-Decoder for Anomaly Detection,” URL: https://arxiv.org/abs/1802.06360, Model: Long Short-Term Memory (LSTM), Dataset: Time Series Anomaly Detection Benchmark, Github: https://github.com/ChunFan-AST/LSTM_Anomaly_Detection

AIMKG responses can include model and dataset for each pipeline. Please refer Table 7 for model related examples. Please refer Supplementary material for more experiments with ChatGPT.

On the other hand, AIMKG returns a list of relevant pipelines for *Supervised Anomaly Detection* tasks as the exact match of the task is unavailable. For brevity, the responses currently include the paper and git repository from which the pipeline can be reproduced. However, AIMKG can also list associated datasets, models, and metrics for some of these pipelines. It is noteworthy that the first recommendation task, *Weakly Supervised Defect Detection*, did not explicitly mention the word *anomaly*. However, our recommender captured that *defect detection* is synonymous with *anomaly detection* in the domain of AI by just using a model pretrained for generic tasks. This also demonstrates the efficiency of embedding and semantic property-based ranking functions described in Equation 2. To add, the AIMKG recommender is explainable by design and the results are explainable.

To summarize, AIMKG produces relevant explainable results and also ensures the reproducibility of the recommended pipelines. While ChatGPT may respond with relevant models for familiar tasks, it hallucinates for many other cases, making it unreliable. Though ChatGPT has access to the data sources AIMKG is constructed with, it cannot construct an AI pipeline from the information available to it. Therefore, the construction of AIMKG enhanced with semantic knowledge is essential to recommend relevant pipelines to users.

## 7 Conclusion

In this study, we proposed Common Metadata Ontology (CMO) to construct an Artificial Intelligence pipeline Metadata Knowledge Graph (AIMKG), a first-of-its-kind knowledge graph for AI pipelines. AI pipeline metadata from open sources such as Papers-with-Code, OpenML, and HuggingFace are integrated to AIMKG, resulting in 1.6 million pipelines with semantic enhancements. The semantic enhancements incorporated in AIMKG capture implicit knowledge (Figure 5) and enhance reasoning capabilities. AIMKG can also store multimodal data types such as embeddings of task, dataset, model and pipeline nodes, supporting text, and numeric and vector data types. Using the computed semantic properties and embeddings, we introduced a custom heuristic ranking metric to rank relevant pipelines for recommendations using task, dataset, or model. The custom heuristic ranking function captured the underlying semantics of the pipeline entities, resulting in more relevant recommendations than the MLSchema-based recommender. The semantic properties also enhance search, as shown in Figure 5. To enable natural language queries for pipelines, we proposed a custom graph embedding aggregation model to retrieve and recommend relevant pipelines. We also demonstrated the potential of AIMKG in optimizing pipelines by seeding them with relevant recommendations. Therefore, AIMKG is an atlas for navigating the rapidly evolving artificial intelligence world.

Currently, not all tasks and datasets in AIMKG have computed semantic properties such as modalities and categories. To address this, we plan to leverage reports and manuscripts associated with pipelines to automatically compute these properties, reducing the biases associated with manually curated vocabularies. In addition, we intend to calculate further semantic and statistical properties for datasets and models, such as dataset image size, color scale, number of classes, data points per class, and model type. We aim to integrate metadata from other open-source repositories, such as Kaggle and the Common Metadata Framework (Koomthanam et al., 2024), into AIMKG. To enrich recommendations and ensure completeness for all 1.6 million pipelines, we plan to utilize fine-tuned language models for extracting information from research papers. Although community-driven sources such as Papers-with-Code, OpenML, and Hugging Face are widely used, they may contain metadata inaccuracies. To improve accuracy and reliability, we will implement robust metadata validation techniques (Soedarmadji et al., 2019; Aggour et al., 2017). In the future, we also envision interfacing AIMKG with large language models (LLMs), enabling users to query pipeline lineage, models, datasets, tasks, and other components through an interactive interface.

## Data Availability

The code repository for AI pipeline Metadata Knowledge Graph can be found at - https://github.com/HewlettPackard/ai-metadata-knowledge-graph. Further inquiries can be directed to the corresponding author.
