# Supplementary material for: Constructing a metadata knowledge graph as an atlas for demystifying AI pipeline optimization
Source: Front Big Data. 2025 Jan 7;7:1476506. doi: 10.3389/fdata.2024.1476506 (PMC11748301; doi:10.3389/fdata.2024.1476506)
Supplement: Supplementary file 1 [file Data_Sheet_1.PDF]

Supplementary Material

1 CONCEPT MAPPING

The mapping of concepts from Papers-with-code, OpenML and Huggingface to Common Metadata Ontology (CMO) can be found in Table S1. This concept mapping is performed for construction of AI pipeline Metadata Knowledge Graph (AIMKG). After the construction, these concepts are enhanced with semantic properties.

Table S1. Mapping of concepts from Papers-with-Code, OpenML and Hugging Face to Common Metadata Ontology. The corresponding concept names of each sources in described in their respective columns.

**Nodes in CMO**	**Papers-with-Code**	**OpenML**	**HuggingFace**
**Pipeline:**AI pipeline executed to solve a Task	Extracted from Papers	Flow	Extracted from Models and Tasks
**Report:** Any published or open-source text document regarding the pipeline implementation	Paper	N/A	Extracted from ArxivID
**Task:** The AI Task for which the pipeline is implemented. Example: image classification	Task	Task type + Dataset	Pipeline Tag
**Framework:** The framework used to implement the pipeline and their code repository	Extracted from Git Repository	Extracted from runs	Library
**Stage:** Various stages of the pipeline such as data preprocessing, training, testing or evaluation	Generated	Generated	Generated
**Execution:** Multiple executions of a given stage in a pipeline	Generated	Runs	Generated
**Artifact:** Artifacts such as model, dataset and metric generated at the end of each execution	Generated	Generated	Generated
**Metric:** Subclass of artifact. The evaluation result of each execution	Evaluation and Results	Extracted from Runs	N/A
**Dataset:** Subclass of artifact. The dataset used in each execution.	Dataset	Dataset	Dataset
**Model:** Subclass of artifact. The model used in each execution or produced as a result of an execution	Method	Extracted from Flow	Model
**Hyperparameters:** Parameter setting using for each execution of a stage	N/A (will be extracted from papers)	Extracted from runs	N/A

2 SENSITIVITY ANALYSIS OF CUSTOM AGGREGATION MODEL

A sensitivity analysis of the proposed model (Cust_Agg_Knowledge) for semantic properties is conducted by randomly assigning semantic properties to pipelines. The hypothesis is that meaningful semantic properties are essential to improve the model's performance. Sensitivity analysis shows that the model's retrieval performance decreased with modified semantic properties, with retrieval@1 dropping from 76.3% to 75.3%, retrieval@3 from 85.4% to 83.2%, and retrieval@5 from 87.7% to 86.6%. The variation is minor since the semantic properties are not extensive at this time of the paper and are available only to certain pipeline components. This indicates that semantic properties can impact retrieval accuracy, particularly at higher retrieval ranks. We plan to curate extensive semantic properties to each pipeline component in our future work.

3 ABLATION STUDY

Tables 4 and 5 also serve as an ablation study to evaluate the effectiveness of the proposed custom heuristic ranking function and the role of semantic properties in the custom aggregation model. Table 4 details the relevance of recommendations produced by the custom heuristic ranking function (AIMKG) and the MLSchema-based knowledge graph (MLS-KG). Notably, the MLS-KG does not include computed semantic properties; therefore, its ranking function relies solely on embedding-based similarity and token matching of task names. The results demonstrate that the proposed custom heuristic ranking function, which combines embedding-based similarity of task names, token-based similarity, and token-based similarity of computed semantic properties, achieved superior performance. This underscores the value of incorporating semantic properties in enhancing recommendation relevance. Table 5 compares the performance of two versions of the proposed model: **Custom_Agg** (trained without semantic properties) and **Custom_Agg_Knowledge** (trained with semantic properties). The model trained with knowledge outperformed its counterpart, emphasizing the positive impact of semantic properties on improving recommendation and retrieval outcomes across both modeling approaches.

4 ANALYSIS ON QUERIES GENERATED BY CHATGPT FOR CUSTOM AGGREGATION MODEL

In this section, we analyze the queries generated by ChatGPT for custom aggregation model. Figure S1 shows the histogram of cosine similarities computed between the pipeline description along with title against query sentences. This is to ensure that the queries generated are relevant to the pipeline and are descriptive of the pipeline. To add, the results from model **Sent_Trans** in Table 5. also captures the relevance through retrieval scores. The distribution shown in Figure S1 seems to be of accepted norm by other sources (MLflow (2024)). Figure S2 shows histrogram of query lengths in terms of number of words. This showcases the variation in query lengths to which our custom aggregation model was able to generalize.

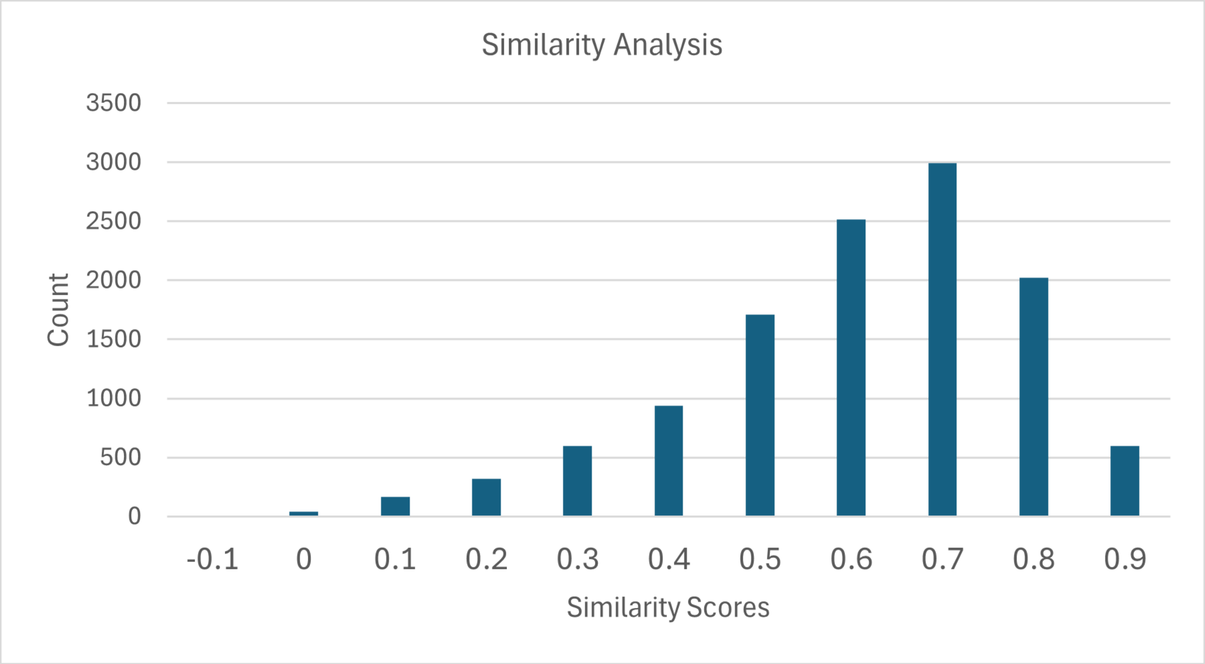

Figure S1. A histogram of cosine similarity metric computed between query and pipline description with title

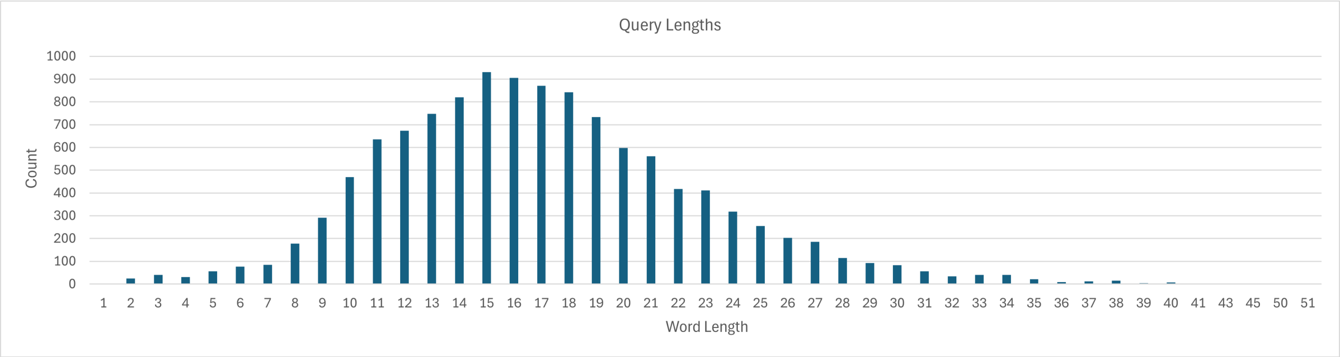

Figure S2. A histogram of length of queries

5 SAMPLE PIPELINES FROM AIMKG

This section includes a few of example pipelines that exists in AIMKG. The intent is to provide an illustration of how pipeline metadata from diverse data sources such as Papers-with-code, OpenML and Huggignface is instantiated by CMO. Figure S3 consists of two pipeline metadata lineage for Bias Mitigation task. Each pipeline executes a different algorithm namely Adversarial Debiasing and Reweighing. For brevity and clarity, the entire pipeline could not be included. The ellipsis represents continuation of metadata lineage of various executions performed for the respective pipelines and stages. Figure S4 represents a pipeline from Huggingface instantiated by Common Metadata Ontology. It illustrates how a pipeline is constructed from one of the data sources. The pipeline is executed for Speech Recognition

task by Wav2Vec model using the datasets Common Voice and Robust Speech Event dataset. Most of the pipelines from Papers-with-code and Huggingface usually reports the results obtained from their test stage. Therefore, these pipelines currently have only test stage.

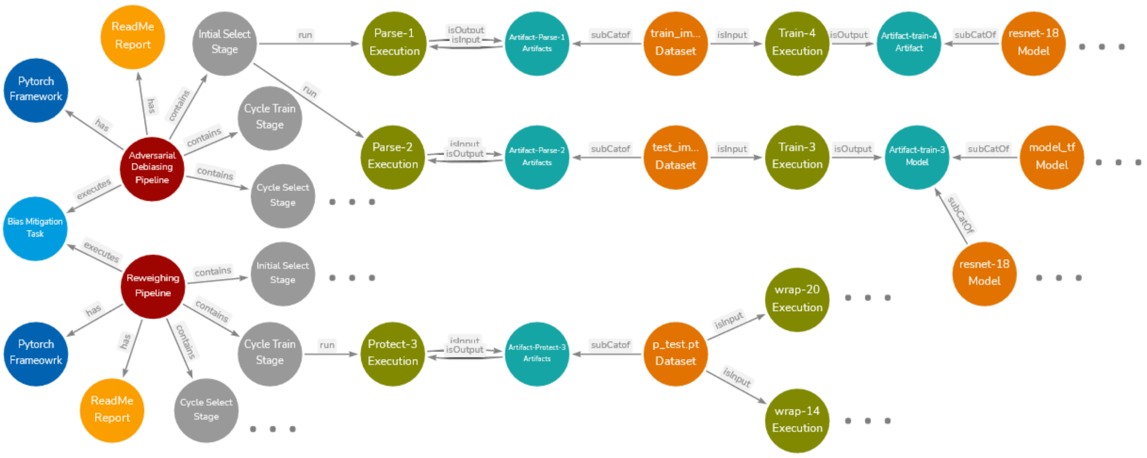

Figure S3. A sample pipeline that exists in AIMKG, instantiated by Common Metadata Ontology. For the Bias Mitigation task (light blue node), two pipelines (red nodes) namely, Adversarial Debiasing and Reweighing have been executed. Each pipeline consists of its own stages, executions, and artifacts. The figure shows a subset of a complex pipeline for brevity.

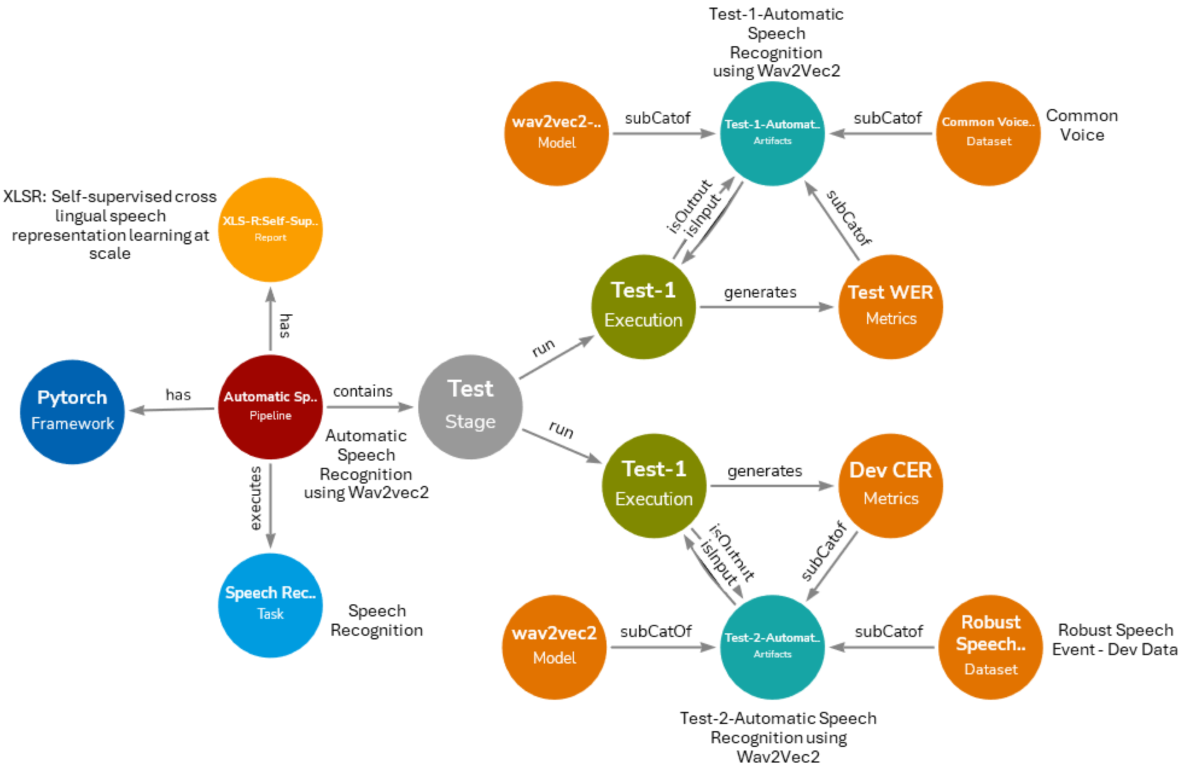

Figure S4. An example pipeline from Huggingface instantiated by Common Metadata Ontology. The pipeline is executed for Speech Recognition task by Wav2Vec model using the datasets Common Voice and Robust Speech Event dataset.

6 EXPERIMENTS WITH CHATGPT

In this section we include additional prompts that we attempted with ChatGPT to recommend relevant pipelines.

6.1 Example-1

We prompted ChatGPT to recommend pipelines for *supervised anomaly detection7* without a sample expected response as mentioned in Table S2. The response obtained from ChatGPT for this query is generic and limited. ChatGPT returned an algorithm or approach that is used for anomaly detection. The response did not include any provenance information such as published paper or code base. The response from ChatGPT also mentions that it cannot name a specific dataset and a model associated with its parameters used for the Supervised Anomaly Detection task. However, ChatGPT did not hallucinate in this experiment unlike when prompted without a sample response.

Table S2. ChatGPT response for pipelines executed for *Supervised Anomaly Detection*

EXAMPLE-1

Prompt: Give me a list of AI pipelines executed for the task Supervised Anomaly Detection with details of dataset name, model name and hyperparameters with their respective paper or git repo.

Response: As of my last knowledge update in January 2022, there isn't a comprehensive and specific list of AI pipelines for Supervised Anomaly Detection that includes detailed information about datasets, model names, hyperparameters, papers, or Git repositories. However, I can provide a generic example of what a supervised anomaly detection pipeline might look like, and you can explore specific papers and repositories for more detailed information:

Example Supervised Anomaly Detection Pipeline: 1. Dataset: Name: Credit Card Fraud Detection Dataset, Description: A dataset with labeled examples of normal and fraudulent credit card transactions.

2. Model: Name: Isolation Forest, Description: An ensemble-based anomaly detection algorithm that isolates anomalies in a dataset.

3. Hyperparameters: Number of Trees: 100, Maximum Samples: 'auto', Contamination: 0.01

4. Paper or Git Repo: Isolation Forest Paper, scikit-learn Isolation Forest

For a more extensive and up-to-date list, you may want to explore academic publications, conferences, or specific machine learning repositories on platforms like GitHub. Search for papers related to supervised anomaly detection, and you can often find details about the datasets used, models implemented, and hyperparameters tuned. Additionally, you can explore well-known anomaly detection frameworks or libraries such as PyOD (Python Outlier Detection) that provide various algorithms for anomaly detection. Each algorithm within these libraries may have its own set of hyperparameters and, in some cases, associated papers or documentation. Keep in mind that the field of anomaly detection is dynamic, and new papers and implementations may have been introduced since my last update. Always refer to the latest literature and repositories for the most current information.

6.2 Example-2

In this is example, we prompted ChatGPT to recommend pipelines for a more familiar task, *3D aware Image synthesis* and the responses are presented in Table S3.In this example, ChatGPT returned a list of paper which are model-centric to the query. That is, it returned papers where models or approaches were introduced that could be applied for the task in the query, 3D aware image synthesis.

Table S3. ChatGPT response for pipelines executed for *3D aware Image synthesis*

EXAMPLE-2

Prompt: Give me a list of AI pipelines executed for the task 3D aware image synthesis with details of dataset name, model name and hyperparameters with their respective paper or git repo.

Response: Creating a comprehensive list of AI pipelines for 3D-aware image synthesis along with specific details, dataset names, model names, hyperparameters, and provenance information is challenging due to the dynamic nature of research in this field. However, I can provide a list of some notable methods along with their associated papers or repositories. Please note that the details may be simplified, and you should refer to the original papers or repositories for precise information. 1. DeepVoxels:

Dataset: ShapeNet, Model: DeepVoxels, Paper: "DeepVoxels: Learning Persistent 3D Feature Embeddings" by Park et al. (2017), Repository: Not specified, Hyperparameters: Not specified 2. NeRF (Neural Radiance Fields):

Dataset: Various scenes or custom datasets, Model: NeRF, Paper: "NeRF: Representing Scenes as Neural Radiance Fields for View Synthesis" by Ben Mildenhall et al. (2020), Repository: https://github.com/bmild/nerf, Hyperparameters: Rays per pixel, network architecture, learning rates. 3. 3D-GAN:

Dataset: ShapeNet, Model: 3D-GAN, Paper: "Learning a Probabilistic Latent Space of Object Shapes via 3D Generative-Adversarial Modeling" by Wu et al. (2016), Repository: https://github.com/zck119/3dgan-release, Hyperparameters: Latent space dimensions, learning rates, batch sizes.

Remember to explore the provided repositories and papers for more detailed information on the models and their implementations. Additionally, keep in mind that the field is evolving, and new methods may have been introduced since the latest information available to me (up to January 2022).

6.3 Example-3

In this example, we prompt the ChatGPT to recommend relevant pipelines for *Sequence speech signal recognition*. In this example, paper-1 is indeed present, and the provided URL is accurate. However, the indicated GitHub link does not lead to the implementation of the paper. Additionally, the generated model and dataset names do not correspond to the artifacts utilized in the paper. Similarly, for paper-2, the paper is present, and the URL is valid, yet the provided GitHub link is associated with the paper but does not lead to its specific implementation. Furthermore, the focus of the paper revolves more around computational enhancements rather than sequence speech signal recognition. As for paper-3, it does not exist, and the specified URL corresponds to a different paper. The GitHub link provided is identical to the one recommended with paper-1. Consequently, the model and dataset details are irrelevant.

Table S4. ChatGPT response for pipelines executed for *Sequence speech signal recognition*

EXAMPLE-3

Prompt: Create a session where you are an AI pipeline recommender. I will request for a pipeline that has been executed in the past. You can use any source you want. For example, when I request to recommend a pipeline for ”3d video object tracking”, I expect a response as follows:

Similar Task Name: 3D Object Tracking Similarity: 0.6083 Piplines: Exploring Point-BEV Fusion for 3D Point Cloud Object Tracking with Transformer, URL: https://arxiv.org/pdf/2208.05216v1.pdf, Model: Transformer, Dataset: KITTI, Github: https://github.com/jasonkks/pttr Piplines: MOTSLAM: MOT-assisted monocular dynamic SLAM using single-view depth estimation, URL: https://arxiv.org/pdf/2210.02038v1.pdf, Model: SLAM, Dataset: KITTI, Github: none

Now give me the pipelines executed for sequence speech signal recognition.

Response:

Similar Task Name: Sequence Speech Signal Recognition, Similarity: 0.6254

Pipelines:

1)Paper Title: ”Listen, Attend and Spell”, URL: https://arxiv.org/abs/1508.01211, Model: Listen, Attend, and Spell (LAS), Dataset: LibriSpeech, Github: https://github.com/espnet/espnet

2) Paper Title: ”Deep Speech 2: End-to-End Speech Recognition in English and Mandarin”, URL: https://arxiv.org/abs/1512.02595, Model: Deep Speech 2, Dataset: Baidu’s Deep Speech Dataset, Github: https://github.com/PaddlePaddle/DeepSpeech

3) Paper Title: ”Attention is All You Need for Chinese Speech Recognition”, URL: https://arxiv.org/abs/2002.09127, Model: Transformer, Dataset: AISHELL-2, Github: https://github.com/espnet/espnet

7 REAL WORLD APPLICATION OF AIMKG

AIMKG has a broader practical impact and can be applied in fields such as healthcare and finance for pipeline optimization through relevant pipeline recommendations. It can also serve as a tool to study the literature and state-of-the-art in these domains. For instance, in healthcare, AIMKG could support image segmentation tasks by recommending optimal combinations of preprocessing, model selection, and evaluation metrics. Similarly, in the finance domain, AIMKG can recommend relevant pipelines for demand forecasting, credit scoring, or any other tasks solved using AI models to support the firm. A few examples of pipeline results for tasks in the Healthcare, Finance, and Legal domains are included in Table S5. Due to data sensitivity or liability issues, the code base may be unavailable for legal or finance domains. The models, datasets, and metrics are available for these pipelines but are not included in the table for brevity.

Table S5. An example demonstrating usage of AIMKG for real-world applications

Domain	Task	Pipelines
Healthcare	Medical Image Segmentation	1.Heart Segmentation From MRI Scans Using Convolutional Neural Network, Paper: `https://arxiv.org/abs/1911.09332v1`, Code: `https://github.com/Shak97/iceest2019`<br>2. Neural Style Transfer Improves 3D Cardiovascular MR Image Segmentation on Inconsistent Data, Paper: `https://arxiv.org/abs/1909.09716v1` , Code: `https://github.com/horsepurve/StyleSegor`<br>3. Multi-Depth Fusion Network for Whole-Heart CT Image Segmentation, Paper: `https://ieeexplore.ieee.org/document/8642875`
Healthcare	Drug Discovery (Protein prediction is vital to drug discovery)	1. Protein Secondary Structure Prediction Using Cascaded Convolutional and Recurrent Neural Networks, Paper: http://arxiv.org/pdf/1604.07176v1.pdf<br>2. Adaptive Residue-wise Profile Fusion for Low Homologous Protein SecondaryStructure Prediction Using External Knowledge, Paper: https://arxiv.org/pdf/2108.04176v1.pdf<br>3. MUFold-SS: Protein Secondary Structure Prediction Using Deep Inception-Inside-Inception Networks, Paper: http://arxiv.org/pdf/1709.06165v1.pdf
Finance	Demand Forecasting in Finance	1. Trimming the Sail: A Second-order Learning Paradigm for Stock Prediction, Paper: http://arxiv.org/abs/2002.06878v1<br>2. Share Price Prediction of Aerospace Relevant Companies with Recurrent Neural Networks based on PCA, Paper: https://arxiv.org/abs/2008.11788v1<br>3. Temporal Relational Ranking for Stock Prediction, Code: `https://github.com/fulifeng/Temporal_Relational_Stock_Ranking`, Paper: http://arxiv.org/abs/1809.09441v1
Finance	Credit Scoring framework in finance	1. Improving a Credit Scoring Model by Incorporating Bank Statement Derived Feature, Paper: http://arxiv.org/abs/1611.00252v2<br>2. Machine Learning approach for Credit Scoring, Paper: https://arxiv.org/abs/2008.01687v1<br>3. A Novel Classification Approach for Credit Scoring based on Gaussian Mixture Models, Paper: https://arxiv.org/abs/2010.13388v1
Legal	Legal Contract analysis	1. Numerical Reasoning over Legal Contracts via Relational Database, Paper: `https://openreview.net/forum?id=a9_4vd4dczF`<br>2. Development of an Enterprise-Grade Contract Understanding System, Paper: https://aclanthology.org/2021.naacl-industry.28
Legal	Risk assessment in legal contracts	1. Use of Artificial Intelligence to Analyse Risk in Legal Documents for Better Decision Support, Paper: https://arxiv.org/abs/1912.01111v1<br>2. Risk measures induced by efficient insurance contracts , Paper: https://arxiv.org/abs/2109.00314v2

8 SYSTEM PERFORMANCE

Neo4j Community version 5.16 serves as the graph data store, while query computations using the custom heuristic ranking function are executed through the Python 3 Neo4j API. The APOC (Awesome Procedures on Cypher) library, an add-on provided by Neo4j for batch parallel loading, allows the entire data to be loaded in 101.03 seconds. A scalability analysis shown in Figure S5 demonstrates the system's capacity to handle the increase in data volume. For custom heuristic ranking functions, embeddings are precomputed for 10k tasks, 53k datasets, and 280k models and stored in an H5PY file. The time taken to generate and store embeddings for tasks, datasets, and models are 80 seconds (1 min 20 secs), 409 seconds (6min 49 secs), and 2220 seconds (36min 40 secs), respectively. The computation of embeddings utilizes a 1xV100 GPU with 40GB RAM. Executing the custom heuristic ranking function for an unknown query and ranking similar tasks, datasets, and models takes 1.07 seconds, 3.04 seconds, and 11.94 seconds, respectively as shown in Figure S6. These computations also rely on a 1xV100 GPU with 40GB of memory. The use of H5PY files for storing and retrieving embeddings significantly enhances performance.

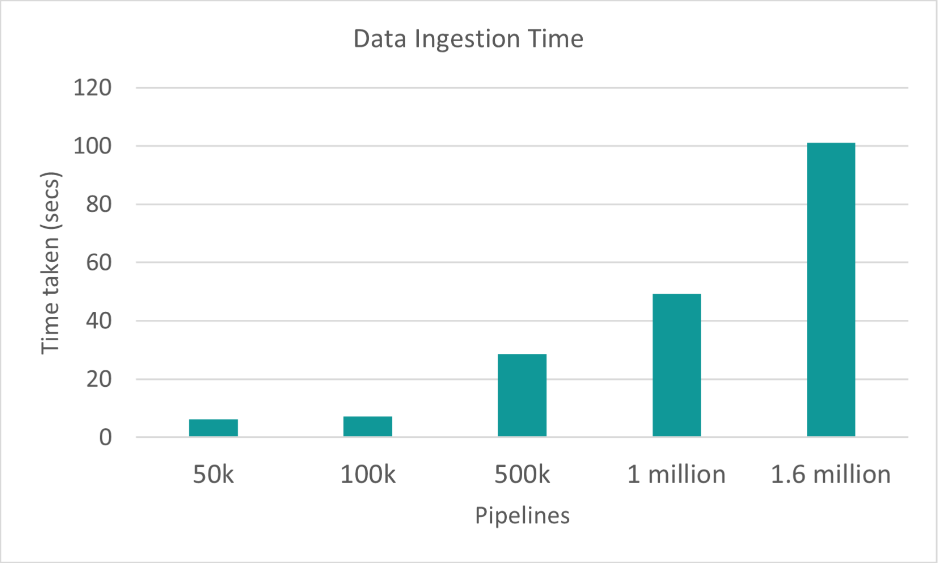

Figure S5. Time taken by the system to ingest data of varying volumes

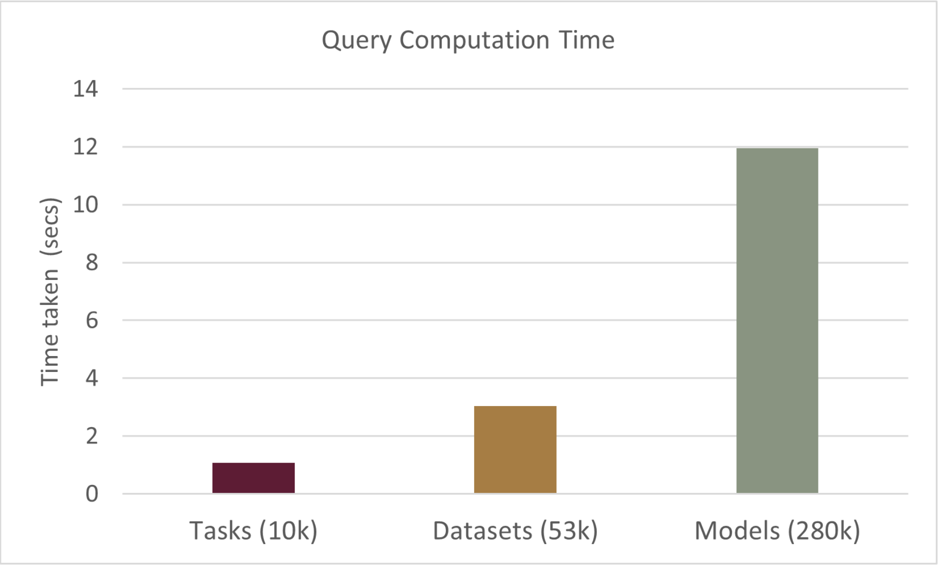

Figure S6. Time taken by the system to compute custom heuristic ranking functions for 10k tasks, 53k datasets, and 280k models

9 SYSTEM MAINTENANCE

The data is periodically retrieved from various sources to gather new information and update existing records, especially when new properties are introduced. It takes 2-3 weeks to gather all the data from Papers-with-code, 1 hour for OpenML and 3 days for Huggingface. If new properties are detected, manual intervention is employed to integrate these properties into CMO and map the data appropriately. Duplicate pipelines from the same sources are eliminated using unique hashes. Each source employs specific fields as primary keys. For instance, paper IDs are used as primary keys in Papers-with-Code, while model IDs and dataset IDs serve as primary keys for Huggingface models and datasets. These primary keys are used to generate unique hashes that help remove duplicates. Duplication across different sources is minimized using the ArXiv IDs associated with papers. Both Papers-with-Code and Huggingface link their papers and models to ArXiv IDs, allowing metadata integration that reduces duplication and enriches the metadata by combining data from multiple sources. Once new data is retrieved, embeddings of node names are computed and stored to facilitate recommendations. Currently, the time taken to generate and store embeddings for tasks, datasets, and models is 80 seconds (1 min 20 secs), 409 seconds (6min 49 secs), and 2220 seconds (36min 40 secs), respectively. The preprocessed data is then mapped to CMO and pushed into Neo4j to create a graph database, from which pipelines or their components can be queried for search and recommendations. However, the main limitation lies in the validation of the pipeline metadata. Although domain experts often contribute to this metadata, there remains a risk of inaccuracies. Identifying and filtering out incorrect data is a significant challenge. To address this, future work will include validation methods leveraging accumulated metadata to improve accuracy and reliability. For instance, incorporating user feedback on metadata during searches could help identify inaccuracies in metadata. In addition, there might be duplicate models in Huggingface or models with very minor variations uploaded by different users. We plan to adopt metadata validation strategies similar to . Moreover, we intend to extract metadata

from published papers or reports to enhance pipeline graphs, ensuring that all pipelines include datasets, models, and metrics as nodes.

10 DATA ANALYSIS

An exploratory data analysis performed on AIMKG is reported in Table S6

Table S6. An example demonstrating usage of AIMKG for real-world applications

	Papers-with-code	OpenML	Huggingface
Pipeline	1029941	3777	267108
Task	3685	6808	18
Report	1027958	NA	467
Framework	116633	2042	8
Stage	1027959	3777	267113
Execution	1027958	673260	287139
Artifact	1027966	673270	287143
Model	1892	12380	267138
Dataset	3366	2705	47316
Metrics	33836	673360	64422

11 ABBREVIATIONS

- AI - Artificial Intelligence
- KG - Knowledge Graph
- NLP - Natural Language Processing
- CMF - Common Metadata Framework
- CMO - Common Metadata Ontology
- AIMKG - AI pipeline Metadata Knowledge Graph
- PWC - Papers-with-code
- HF - Huggingface
- R@1: Retrieval at rank 1 (Retrieval@1)
- R@3: Retrieval at rank 3 (Retrieval@3)
- R@5: Retrieval at rank 5 (Retrieval@5)

12 APPENDIX

- CMF - Common Metadata Framework. A pipeline-centric metadata logging framework that tracks the lineages for artifacts and executions of distributed AI pipelines. It provides APIs to record and query the metadata associated with ML pipelines.
- CMO - Common Metadata Ontology is an ontology designed to semantically enrich the metadata used in AI pipelines. It is constructed based on the principles of the Common Metadata Framework.
- AIMKG - AI pipeline Metadata Knowledge Graph, constructed by integrating metadata from various open sources such as Papers-with-code, OpenML, and Huggingface
- Papers-with-code - One of the three data sources of AIMKG. It is an online resource that connects academic research papers with their corresponding code implementations.
- OpenML - One of the three data sources of AIMKG. It is an open platform for sharing, organizing, and collaborating on machine learning experiments, datasets, and models, promoting transparency and reproducibility in data science research.
- Huggingface - One of the three data sources of AIMKG. It is an open-source platform that provides datasets and models for AI research, enabling developers to access, share, and deploy state-of-the-art models.
- Graph Data Models - The graph structure of each source is constructed from their Relational Database format.
- R@1: Retrieval at rank 1 (indicating the model's ability to retrieve relevant items within the top 1st position correctly)
- R@3: Retrieval at rank 3 (indicating the model's ability to correctly retrieve relevant items within the top 1, 2, or 3 positions)
- R@5: Retrieval at rank 5 (indicating the model's ability to correctly retrieve relevant items within the top 1, 2, 3, 4, or 5 positions)

REFERENCES

[Dataset] MLflow (2024). Question generation for retrieval evaluation. Accessed: 2024-07-27
